# Bioactivity Studies of *β*-Lactam Derived Polycyclic Fused Pyrroli-Dine/Pyrrolizidine Derivatives in Dentistry: *In Vitro*, *In Vivo* and *In Silico* Studies

**DOI:** 10.1371/journal.pone.0131433

**Published:** 2015-07-17

**Authors:** Gowri Meiyazhagan, Rajesh Raju, Sofi Beaula Winfred, Bhavani Mannivanan, Hemadev Bhoopalan, Venkatesh Shankar, Sathiya Sekar, Deepa Parvathi Venkatachalam, Ravishankar Pitani, Venkateshbabu Nagendrababu, Malini Thaiman, Kandaswamy Devivanayagam, Jeyakanthan Jayaraman, Raghunathan Ragavachary, Ganesh Venkatraman

**Affiliations:** 1 Department of Human Genetics, Sri Ramachandra University, Porur, Chennai, Tamilnadu, India; 2 Department of Organic Chemistry, University of Madras, Guindy Campus, Chennai, Tamilnadu, India; 3 Department of Bioinformatics, Alagappa University, Karaikudi, Tamilnadu, India; 4 Center for Toxicology and Developmental Research, Sri Ramachandra University, Porur, Chennai, Tamilnadu, India; 5 Department of Community Medicine, Sri Ramachandra University, Porur, Chennai Tamilnadu, India; 6 Department of Conservative Dentistry and Endodontics, Sri Ramachandra University, Porur, Chennai, Tamilnadu, India; 7 Central Research Facility, Sri Ramachandra University, Porur, Chennai, Tamilnadu, India; Institut Pasteur Paris, FRANCE

## Abstract

The antibacterial activity of *β*-lactam derived polycyclic fused pyrrolidine/pyrrolizidine derivatives synthesized by 1, 3-dipolar cycloaddition reaction was evaluated against microbes involved in dental infection. Fifteen compounds were screened; among them compound 3 showed efficient antibacterial activity in an *ex vivo* dentinal tubule model and *in vivo* mice infectious model. *In silico* docking studies showed greater affinity to penicillin binding protein. Cell damage was observed under Scanning Electron Microscopy (SEM) which was further proved by Confocal Laser Scanning Microscope (CLSM) and quantified using Flow Cytometry by PI up-take. Compound 3 treated *E*. *faecalis* showed ROS generation and loss of membrane integrity was quantified by flow cytometry. Compound 3 was also found to be active against resistant *E*. *faecalis* strains isolated from failed root canal treatment cases. Further, compound 3 was found to be hemocompatible, not cytotoxic to normal mammalian NIH 3T3 cells and non mutagenic. It was concluded that *β*-lactam compound 3 exhibited promising antibacterial activity against *E*. *faecalis* involved in root canal infections and the mechanism of action was deciphered. The results of this research can be further implicated in the development of potent antibacterial medicaments with applications in dentistry.

## Introduction

Dental infections are prevalent forms of microbial infections in humans and have considerable implications on health and well being of the individuals. Among the dental infections, inflammation and infection of the root canal are common among all age groups [1]. Currently, the management of root canal infections involves cleaning the canal with powerful irrigants like chlorhexidine, sodium hypochlorite, etc., followed by intracanal medicament application with calcium hydroxide [2]. Though these treatments have been in vogue for a long time, root canal failures do occur [3, 4]. Root canal treatment failures have been primarily attributed to the presence of a resistant form of *Enterococcus faecalis*, which has been isolated as the single organism in treatment- failure cases [2, 5]. Hence, the objective of the present study was to screen the new *β-*lactam compounds for their activity against the microbes involved in root canal infections and root canal failures.

The bioactive moiety *β-*lactam present in commonly used antibiotics like penicillin, cephalosporins, carbapenems, nocardicins and monobactams can be easily synthesized by organic synthesis [6–8]. The bioactivity of *β-*lactams is not only restricted to antimicrobial action but has also been shown to extend to inhibition of absorption of cholesterol, human cytomegalovirus protease inhibition and thrombin inhibition [9–11]. The substituted hydroxyl *β-*lactams have been used in semi- synthetic process for the preparation of useful anticancer compounds like paclitaxel (Taxol) and docetaxol (Taxotene) [12, 13]. Owing to its varied bioactivities, the authors and their research groups have been actively involved in the synthesis of *β*-lactam derived heterocycles and some of the compounds were identified to possess good bioactivity. Recently, *β*-lactam substituted polycyclic fused pyrrolidine and pyrrolizidine derivatives were developed by reacting *β-*lactam Baylis-Hillman Adduct (BHA) as a dipolarophile with azomethineylide generated from di/tri-ketones and secondary amino acids through [3+2]-cycloaddition reaction [14, 15].

Herein, the authors have attempted to describe the antibacterial activity of these polycyclic fused pyrrolidine and pyrrolizidine derivatives against *E*. *faecalis* and other pathogens in root canal infection. The anti-microbial efficacy of the compounds were evaluated on an *ex vivo* tooth and *in vivo* mice model to ascertain the ultimate application of these compounds. Since these compounds are intended for human application, they were also tested for their cytotoxicity and biocompatibility properties.

## Materials and Methods

### Chemistry

#### Experimental section

All melting points were uncorrected. The progression of all the reactions was monitored by thin layer chromatography (TLC) using hexanes/ethyl acetate mixture as eluent. Column chromatography was carried out on silica gel by using increasing polarity. ^1^H, ^13^C and DEPT-135 spectra were recorded in CDCl_3_ using TMS as an internal standard on a Bruker 300 MHz spectrometer at room temperature. Chemical shift values were quoted in parts per million (ppm) and coupling constants (J) were quoted in Hertz (Hz). Mass spectra were recorded on JEOL GC mate mass spectrometer. The X-ray diffraction measurements were carried out at 298 K on a Bruker (2008) SMART APEX 2 area detector diffractometer (S1 File).

### Pharmacological studies

#### Preparation of microbial suspension

The antibacterial activity of *β-*lactam compounds was tested against *E*. *faecalis* (ATCC 29212), *S*. *aureus*, *Streptococcus* sp and five resistant *E*. *faecalis* strains (RS1-5) which were isolated from root canal treatment failure cases. The strains thus isolated were found to be resistant to ampicillin and calcium hydroxide (intracanal medicament). Purity of isolate was tested based on the phenotypic (Biochemical test) and genotypic (Sequencing) tests. The bacterial cultures were diluted and adjusted to cell suspension of 10^6^ colony forming unit (CFU) per ml by measuring optical density (O.D) at 600 nm using spectrophotometer.

#### Preparation of *β*-lactam derivatives for bioactivity testing

Fifteen *β-*lactam derivatives synthesized by cycloaddition reaction were used for screening and were dissolved in dimethyl sulfoxide (DMSO) for all the studies.

#### Determination of zone of inhibition

The well diffusion method was used to determine the antibacterial activity of *β-*lactam compounds against *E*. *faecalis* (ATCC 29212), *S*. *aureus*, *Streptococcus* sp and five resistant *E*. *faecalis* strains (RS1-5) on BHI agar plates. Diluted inoculum was spread on the surface of the plates and after 5 min; wells were drilled using well borer under aseptic conditions. Each well was loaded with 100 μg of each *β-*lactam compound. Ampicillin was used as reference compound and 100 μl of DMSO served as vehicle control. The plates were incubated for 24 to 48 h at 37°C. The width of the zone of inhibition was measured to evaluate the antibacterial activity of the *β-*lactam compounds against the tested organisms. The experiments were done in duplicates.

#### Determination of minimum inhibitory concentration (MIC)

MIC of *β*-lactam compounds against *E*. *faecalis* (ATCC 29212), *S*. *aureus*, *Streptococcus* sp and five resistant *E*. *faecalis* strains (RS1-5) were determined by micro-dilution method using tri phenyl tetrazolium chloride (TTC) as an indicator. BHI broth was used to culture the test organisms in 96 wells plate. A stock solution of 2 mg of compound was prepared in 10 μl of DMSO. From the stock, 200 μg of each compound was serially diluted by two fold dilution to obtain the concentration range from 200 μg/ml- 0.9 μg/ml. After the dilution, 20 μl of culture (1x 10^6^) was added and the plate was incubated at 37°C for 24 h. After incubation, 50 μl of 0.1% TTC was added and incubated for 3 to 4 h. The color change (from yellow to pink) of broth indicated the growth of organism. After incubation, the O.D was measured at 600 nm using thermoscan spectrophotometer. The experiments were done in triplicates.

#### Time kill assay

*In vitro* time kill assay [16] was performed by diluting an overnight culture of *E*. *faecalis* in BHI to reach the final concentration of 1x10^6^ CFU/ml. The compounds were added according to their MICs and incubated at various time intervals (1 h, 3 h, 6 h, 12 h and 24 h). After incubation, 10 fold serial dilutions were performed for treated and untreated cultures. 100 μl of each sample was plated onto the BHI agar plates which were incubated at 37°C for 24 h. The inhibitory effect was calculated based on the CFU.

### Antibacterial efficacy in an *ex vivo* dentine model

#### Preparation of blocks

The antibacterial efficacy study on dentinal tubules was performed with certain modifications [17]. The protocol was approved by Institutional Ethics Committee of Sri Ramachandra University (IEC-NI/11/DEC/26/81). Single rooted human mandibular premolar teeth extracted from patients who had periodontal or orthodontic treatment were selected. After extraction, the external surface of the tooth was cleaned and immersed in 0.5% NaOCl for 24 h to disinfect the surface of tooth and samples were stored in 0.9% sterile physiological saline at room temperature until use. For the present study, the crown of all tooth samples were cut to obtain the long axis of the teeth using cylindrical bur in high speed hand piece and the remaining middle portion was used. From the root canal, the pulp remnants were removed and used for all studies.

#### Contamination of blocks

All the tooth samples were sterilized at 121°C for 15 min and were placed in BHI broth containing *E*. *faecalis* suspension and incubated for 21 days at 37°C. Three specimens were not incubated with organisms and served as negative control to confirm the sterility of the tooth sample. All the specimens were monitored during this period and the broth was changed every day. After the incubation, the specimens were washed with saline to remove the excess culture from surface of the specimen.

The inoculated samples (n = 40) were grouped as follows: Group 1- Tooth samples treated with *β*-lactam compound 3 (25 μg/ml), Group 2- Tooth samples treated with *β*-lactam compound 7 (25 μg/ml), Group 3- Tooth samples treated with positive control, ampicillin (6 μg/ml) and Group 4- Tooth samples without any treatment which served as negative controls. The compounds 3, 7 and ampicillin were mixed with methyl cellulose and packed onto the *E*. *faecalis* contaminated dentinal blocks and incubated for 24 h at 37°C. After the incubation, the dentinal chips were harvested at two different depths at 200 μm and 400 μm using gates glidden drill. The harvested dental chips were used to quantify the bacterial load by using colony forming units (CFU/mg of dentin). The O.D of the broth from the treated and untreated samples was measured at 600 nm using spectrophotometer and the O.D values were plotted against blank to show the bacterial load.

#### *In vivo* efficacy of *β-*lactam compound

In the present study, 6 weeks old female Balb/C mice were used to determine the antibacterial efficacy of *β-*lactam compound 3 against *E*. *faecalis* [18]. Total of 30 mice (25 g) were used for the study and the protocol was approved by Institutional Animal Ethics Committee of Sri Ramachandra University (IAEC/XXXIII/SRU/260/2013). 100 μl of 4x10^8^ cells/ml of *E*. *faecalis* cells were injected intravenously through tail vein. After 4 h of injection, mice were subcutaneously treated with 25 and 50 mg/kg of *β-*lactam compound 3 and 25 mg/kg of ampicillin. The compound was administered once daily for three days. All the mice were sacrificed after 120 h of bacterial injection. Heart, lungs, brain, liver, kidney, spleen and blood were collected aseptically. For bacterial load reduction, organs were homogenized in 5 ml of sterile phosphate buffer saline (PBS). From that 100 μl of aliquot was serially diluted upto 10^6^ dilution. From each dilution 100 μl of diluents was plated onto BHI agar plates. The plates were incubated at 37°C for 24 to 48 h. After incubation, bacterial load reduction in each organ was calculated based on CFU count. For histopathology, organs were fixed with 10% formalin and organ sections were prepared and stained with eosin and hematoxylin. The slides were examined and images were obtained at 400X magnification.

#### Covalent docking calculation

The penicillin binding protein (PDB: 2Z2M) crystal structure complexed with cefditoren was derived from *Streptococcus pneumoniae* strain obtained from protein data bank and this protein was used for docking study [19, 20]. The target protein was subjected to the protein preparation wizard in Maestro [21]. The 2D structures of all fifteen *β*-lactam compounds were drawn by using Marvin-Sketch 5.6 and subsequently prepared by LigPrep module in Maestro [22]. The CovDock docking program from the Maestro [23] was used as in matter of fact that PBP consists of a covalently bound Cefditoren. CovDock utilizes the Glide docking and prime optimization to predict the binding mode of covalent inhibitors, which is used in determining the binding affinity of synthesized fifteen *β*-lactam compounds. To define the covalently bound reactive residue, Ser337 was chosen in the receptor [19]. The centroid of workspace ligand was specified for generating the grid box. The covalent reaction type *β*-lactam addition method was selected. The reaction defines the functional group in the ligand that participates in the interaction with the residue describing the physico-chemical property of that residue and the atom in the ligand that binds to the receptor. The ranking of compounds by the empirical scoring function within program estimates the apparent affinity of a covalent inhibitor and it is reported as the average of the pre-reacted and post-reacted Glide score for a given pose. At the end of covalent docking calculation, five least energy ligand poses were obtained [23, 24].

#### Qualitative and quantitative biofilm assay

For qualitative biofilm assay [25], *E*. *faecalis* cells were seeded on the Whatmann No.1 filter paper and incubated for 48 h at 37°C. After incubation, the filter paper strips were treated with *β*-lactam compound 3 at a concentration equal to MIC for 16 h and strips were immersed in the neutralizing broth to remove residual compounds. The treated strips were observed for their bacterial load reduction. Images were acquired using SEM (Supra 55, Carl Zeiss) at 100KX magnification. Untreated strips served as negative controls. For quantification, biofilm formation assay was performed by microtitre plate assay [26]. Briefly, the 200 μl of *E*. *faecalis* cell suspension was dispensed into the sterile flat bottomed 96 well polystyrene microtitre plate. The plate was incubated at 37°C for 96 h to allow the biofilm formation. After the biofilm formation, the media were decanted and 200 μl of fresh medium was added. *β*-lactam compounds (3, 7 and 6a) were added according to their MICs. Untreated wells served as negative controls. The plate was incubated at 37°C for 24 h. After incubation, the broth was discarded; wells were washed three times with 200 μl of PBS and were fixed with 200 μl methanol for 30 min. After fixation, biofilms were stained with 0.1% crystal violet solution in water for 45 min and wells were washed three times with distilled water to remove the excess stain and air dried. To quantify the biofilm, 200 μl of ethanol/acetone (90:10) was added to each well to destain the biofilms. The O.D of the solubilized crystal violet was measured at 570 nm in a spectrophotometer. The experiments were performed in triplicates.

#### Bacterial viability assay in *ex vivo* dentine model

The tooth samples were inoculated with *E*. *faecalis* for 21 days [27]. After inoculation, the samples were treated with *β*-lactam compound 3 (25 μg/ml). Ampicillin served as reference compound and tooth samples without any treatment served as negative control. After incubation, the tooth samples were sliced using discs. The tooth sample slices were observed for bacterial viability by staining the samples with FDA and PI. Stained samples were observed under CLSM. Tooth sample was observed for live/dead bacterial count and bacterial viability was calculated.

#### Scanning Electron Microscopy

Overnight *E*. *faecalis* cells were seeded on the Whatmann No.1 filter paper and incubated for 24 h at 37°C. After incubation, filter paper strips were treated with *β*-lactam compound 3 at its MIC for 1 h. Untreated strips served as negative controls. The strips were washed three times in 0.1 M phosphate buffer (pH 7.4) and cells were fixed with 2.5% gluteraldehyde for 15 min and dehydrated with ethanol gradient. The sample was coated with gold (15 min) and the images were acquired using SEM (Supra 55, Carl Zeiss) at 100KX magnification [28].

#### Measurement of reactive oxygen species (ROS) production

Amount of ROS generation was measured by flow cytometry analysis with 2, 7-dichlorofluorescein diacetate (DCFH-DA) as described earlier [28]. Briefly, overnight *E*. *faecalis* cells were sub-cultured in BHI broth and adjusted to 1x10^6^ cells /ml. The cells were harvested at 3000 g for 10 min. The cell pellet was then re-suspended in PBS and treated with compound 3 (25 μg/ml) and ampicillin (6 μg/ml) alone and in combination with ascorbic acid (100 μg) for 1 h at 37°C. Untreated cells served as negative controls. Hydrogen peroxide (H_2_O_2_) was used as positive control. After incubation, 10 μM of DCFH-DA in PBS was added and incubated for 10 min at room temperature. After the incubation, the cells were subjected to Fluorescence Activated Cell Sorter (FACS) analysis.

#### PI uptake

Overnight *E*. *faecalis* cells were sub-cultured in BHI broth and adjusted to 1x10^6^ cells /ml and were harvested by centrifugation at 3000 g for 10 min. The pellet was washed and re-suspended in HEPES buffer (5 mM glucose, 5 mM HEPES). The cells were treated with *β*-lactam compound 3 (25 μg/ml) for 45 min at 37°C. Untreated cells served as negative control and ampicillin treated cells served as positive control. After incubation, 5 μl of PI (1 mg/ml) was added and incubated for 15 min at room temperature. After incubation, the cells were subjected to FACS analysis [28].

#### Hemolytic assay

Hemolytic assay [29] was performed using the heparinized 1 ml whole human blood collected from a donor (IEC-NI/11/DEC/26/81) and the RBC pellet was obtained by centrifuging at 1000 g for 5 min. Supernatant was discarded and the RBC pellet was washed with PBS. 1% RBC suspension was prepared and 100 μl of 1% RBC suspension was incubated with *β*-lactam compounds (3, 6a and 7) concentration equal to their MICs and ampicillin for 2 h. After the incubation, the samples were centrifuged at 1000 g for 5 min. Supernatant was collected and O.D was measured at 545 nm to determine the RBC lysis. Supernatant from RBC treated with triton X-100 served as the positive control and untreated RBC with PBS served as negative control. Percentage of lysis was calculated using the formula.

% of hemolysis = [(O.D of test – O.D of negative control) / (O.D of positive control− O.D of negative control)] X 100

#### Mammalian cell cytotoxicity

The cytotoxicity of *β-*lactam compounds were determined by MTT assay on mouse fibroblast cell line (NIH 3T3 obtained from National Centre for Cell Science, Pune) in 96 wells plate [30]. The cells (2x10^4^) were cultured in Dulbecco’s Modified Eagles Medium (DMEM) with 10% fetal bovine serum at 37°C in 5% CO_2_. After incubation, the cells were treated with various concentrations of *β*-lactam compounds (25, 50, 100 and 200 μg/ml) and incubated for 24 h at 37°C. Further, 20 μl of MTT solution (5 mg/ml in PBS) was added after 24 h and incubated for 3 to 4 h at 37°C to allow the formazan product formation by MTT reduction. After incubation, the media was decanted and formazan crystals were dissolved by adding 200 μl of DMSO to obtain the purple colored product. 1% triton X-100 was used as positive control and untreated cells served as negative controls. The O.D was measured at 570 nm and 690 nm and percentage of viability was calculated using the formula.

% of viability= [(O.D of treated cells)/ (O.D of untreated cells)] X100

### *In vitro* genotoxicity of *β*-lactam compounds

#### Bacterial reverse mutation test

To determine the mutagenic property of *β*-lactam compounds, the AMES test was performed using *Salmonella typhimurium* strains TA 98 and TA 100 (His^-^) without metabolic activation system [31]. For pre-incubation method, overnight culture of strains were inoculated into nutrient broth (oxoid broth) and subcultured to obtain 1x10^9^ cells/ml. 100 μl of culture and 10X MIC of *β*-lactam compounds (3 and 7) were added in a tube containing 0.5 ml of phosphate buffer (pH 7.4) and incubated at 37°C for 20 min. After incubation, 2 ml of soft agar which contains 0.5 mM of histidine/biotin was added. The tubes were mixed and poured onto minimal glucose agar plates, the plates were incubated for 48 h and the revertant colonies were counted manually. 0.1% DMSO was used as vehicle control; mitomycin C (5 μg) and sodium azide (2 μg) were used as positive controls. The experiments were done in triplicates.

#### Micronucleus assay

Blood sample was obtained by venipuncture in a heparinized vaccutainer from healthy volunteers after getting their written consent. The protocol was approved by Institutional Ethics Committee of Sri Ramachandra University (IEC-NI/11/DEC/26/81). In a culture flask 80% of RPMI 1640 medium with 20% of FBS, 1 ml of the peripheral blood along with compounds (3 and 7) at their 10X MICs and 40 μg/ml of phytoheamagglutinin (PHA) were added. The cultures were incubated at 37°C for 72 h. At the 44^th^ h, cells were blocked before entering into a cytokinesis using cytochalasin-B (4 μg/ml) and further incubated for 24 h at 37°C. At the end of 72^nd^ h, the cells were treated with hypotonic (0.45%) solution and fixed with carnoy’s fixative. 0.1% DMSO served as vehicle control; bleomycin served as positive control. The slides were casted, stained with giemsa and analyzed. Micronucleus (MN) frequency was calculated using the formula [32].

MN Frequency = Total number of aberration /Total number of cells scored

Standard Error =Square root of (number of aberration) /Total number of cells scored

#### Chromosomal aberration assay

In a culture flask, 80% of RPMI 1640 medium with 20% of FBS, 1 ml of the peripheral blood (IEC-NI/11/DEC/26/81) along with compounds (3 and 7) at their 10X MICs and 40 μg/ml of PHA were added. The cultures were incubated at 37°C for 46 h. At the end of 46^th^ h, the cells were blocked at metaphase stage by adding 0.01% of colchicine. The cultures were further incubated for 2 h. At the end of 48^th^ h, the cells were treated with hypotonic (0.075M) solution and fixed with carnoy’s fixative. The slides were casted and stained with giemsa. Analysis and documentation were performed. Chromosomal aberration (CA) frequency was calculated using the formula mentioned below [33].

CA Frequency = Total number of aberration / Total number of metaphase scored

Standard Error = Square root of (number of aberration) / Total number of metaphase scored

#### *In vivo* genotoxicity of *β*-lactam compound

The *in vivo* genotoxicity of the synthesized *β-*lactam compound was tested in *Drosophila melanogaster* using Somatic Mutation and Recombination Test (SMART). A cross was established by mating 40 virgin flr^3^ females with 20 mwh males in corn meal agar. The parent flies were removed from the bottle post 72 h and the third instar transheterozygous larvae (mwh+/+flr^3^) were collected in PBS containing 1% sucrose. The larvae were transferred to vials with dry *Drosophila* instant medium wetted with sterile water mixed with various concentration of *β-*lactam compound 3 (250 and 500 μg/ vials). 5 mM ethyl methane sulfonate (EMS) was used as positive control while sterile water served as negative control and 0.1% DMSO served as vehicle control. The vials were incubated at 22°C ± 1°C till the larvae moulted to adults by feeding on the compound in the medium. This serves as the source of exposure. These larvae were monitored daily for viability. The adult flies that emerged were collected and anesthetized by cooling (-20°C for 30 min), the wings were detached carefully and mounted on clean glass slides with DPX. The wings were scored for spots under 100X magnification [34].

### Statistical analysis

The values were represented as mean ± SD for time kill assay, hemolytic and cytotoxic assay. Statistical significance was evaluated by non parametric (Kruskal- Wallis) test to check differences in the bacterial load reduction at two depths in an *ex vivo* dentinal tubule model and non parametric (Mann Whitney) test was performed for *in vivo* antibacterial efficacy. Dunnett t (2-sided) test was performed to check statistical significance in wing spot assay. Values less than 0.05 (p < 0.05) was considered to be statistically significant.

## Results

### Chemistry

**Fig 1:**
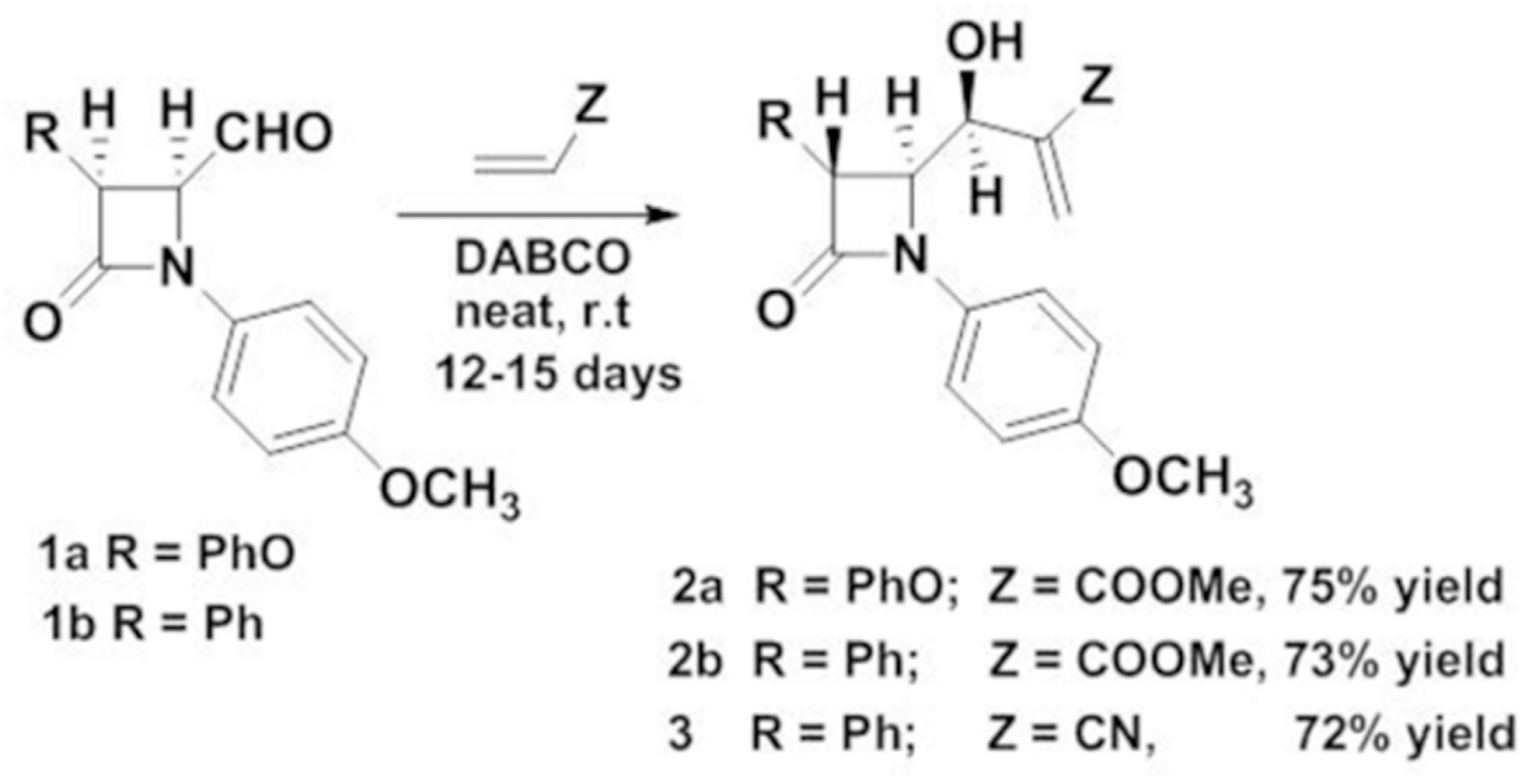
Synthesis of *β-*lactam Baylis-Hillman adducts.

The required precursor, Baylis-Hillman (BH) adduct 2a, 2b and 3,used as a dipolarophile, was synthesized by BH reaction of 4-oxoazetidine-2-carbaldehydes 1a, 1b [35] and methyl acrylate or acrylonitrile in the presence of DABCO as a catalyst (Fig 1). During Baylis-Hillman reaction *β-*lactam aldehydes 1a, 1b, underwent isomerisation in the presence of base DABCO [36] to form more stable trans BH adduct (2a, 2b and 3) as evidenced by X-ray analysis [37]. The BH adduct so prepared was reacted with azomethine ylide, generated from di-/tri-ketones with various secondary amino acids. Thus, 1,3-dipolar cycloaddition reaction of azomethine ylide derived from isatin/acenaphthequinone/ninhydrin and sarcosine/proline with BH adduct 2a/2b/3 as a dipolorophile in refluxed methanol gave *β-*lactam substituted polycyclic heterocycles in good yields.

**Fig 2:**
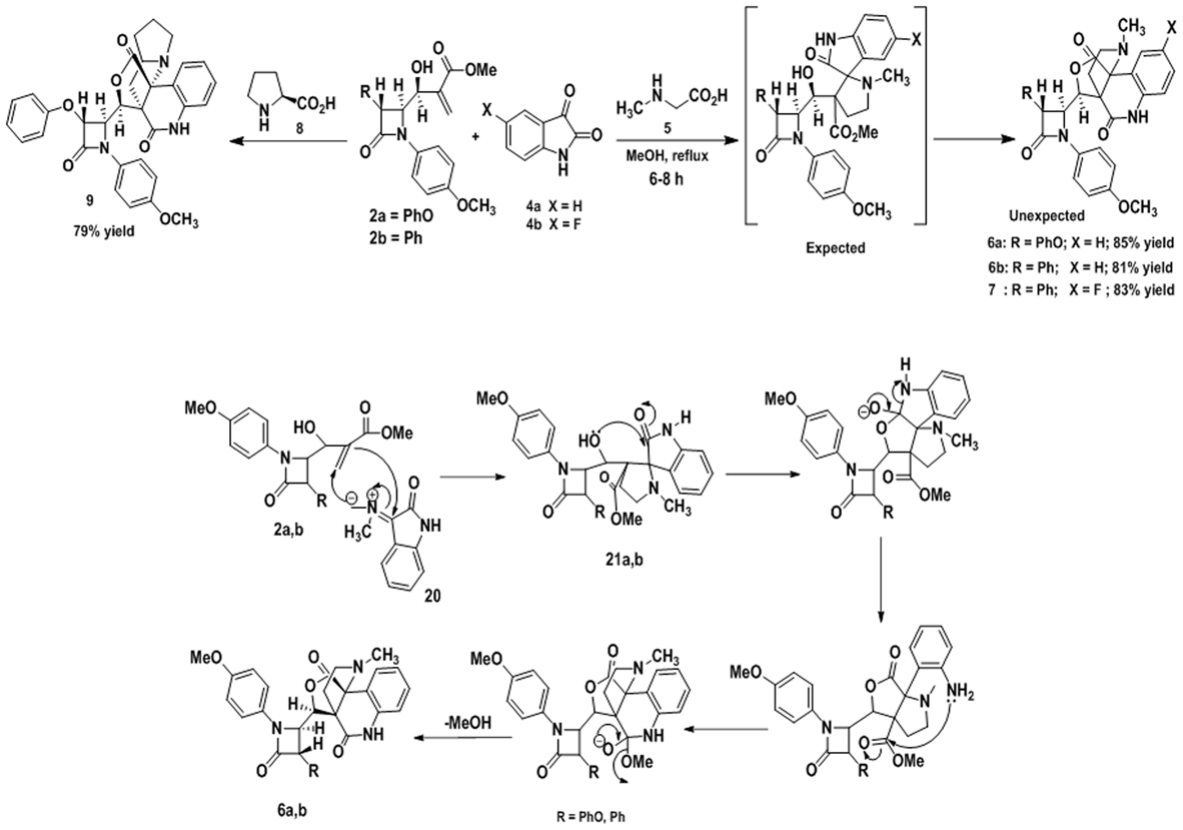
Synthesis of *β-*lactam substituted polycyclic fused pyrrolidine/pyrrolizidine cycloadducts.

The azomethine ylide generated from isatin/sarcosine and isatin/proline reacted with *β-*lactam substituted BH adducts derived from methylacrylate 2a, 2b gave unexpected fused polycyclic pyrrolo/pyrrolizino quinolinone derivatives in good yield (Fig 2). The reaction did not yield the expected spirocycloadduct, but underwent rearrangement by ring opening and ring expansion sequences to yield the polycyclic bridged heterocyclic compound 6a/6b/7 in good yields. A tentative mechanism was proposed to explain the formation of quinolinone derivatives (Fig 1). It was assumed that the compounds 6a and 6b were formed in a one-pot reaction via the sequential 3+2 cycloaddition of azomethine ylide 20 derived from isatin and sarcosine to the Baylis-Hillman adduct 2a/2b as a dipolarophile to give expected cycloadduct 21. Nucleophilic attack on the imido carbonyl of isatin by the hydroxyl group of 21 resulted in the formation of furan ring. The oxygen anion formed gets stabilized by opening of isatin ring to give an aniline derivative. The amino group underwent cyclization by nucleophilic attack on ester carbonyl and subsequent elimination of methoxy group to give the final product 6a/6b (Fig 1). One of the noteworthy observations was the structure of the products 6a/6b/7, which showed that three rings (lactone, pyrrolidine and lactam rings) were fused in single C-C junction.

Similarly the reaction of azomethine ylides generated from other diketone, acenaphthenequinone and triketone ninhydrin with BH adducts 2a, 2b and 3. However, the authors found a different type of product being formed with these di/tri-ketones. In these reactions, the hydroxyl groups of the cycloadduct initially formed underwent intramolecular hemiacetal cyclization with the neighboring carbonyl group to give furanopyrrolidine 11, 12, 16, 17 and furanopyrrolizidine 13, 14, 18, 19 derivatives in good yield.

The structures of the cycloadducts were confirmed by various spectroscopic techniques like ^1^H and ^13^C NMR, mass, IR and proved unambiguously by single crystal X-ray analysis (S1 File).

### Pharmacological studies

#### Antibacterial activity of *β*-lactam compounds

In the present study, antibacterial activities of fifteen synthesized *β*-lactam derivatives were evaluated *in vitro* against the organisms mentioned earlier. Among the fifteen compounds tested, 2a, 3, 6a, 7, 9, 11b, 12, 13a, 13b, 14, 18a, 18b and 19 showed activity against *E*. *faecalis* (ATCC 29212). Compounds 2a, 3, 6a, 7, 9, 11b, 12, 13a, 13b, 14, 18a, 18b and 19 showed activity against resistant *E*. *faecalis* (RS1) while compounds 3, 6a, 7, 9, 11a, 11b, 12, 13a, 13b, 14, 18b and 19 showed efficacy against *E*. *faecalis* (RS2). Compounds 3, 6a, 7, 11b, 12, 13b, 14, 18b and 19 showed activity against *E*. *faecalis* (RS3) and compounds 2a, 3, 6a, 7, 9, 11b, 12, 13a, 14, 16a, 18b and 19 showed activity against *E*. *faecalis* (RS4). Compounds 2a, 3, 6a, 7, 11a, 11b, 12, 13b, 16a, 18b and 19 showed activity against *E*. *faecalis* (RS5) while compounds 2a, 3, 6a, 7, 11b, 12, 13a, 13b, 14, 18a, 18b and 19 showed activity against *Streptococcus* sp and compounds 2a, 3, 6a, 7, 11b, 12, 13b, 14, 16a, 18a and 18b showed activity against *S*. *aureus*. Among them, compound 3 showed activity against all the tested microbes. For *E*. *faecalis*, compound 11b showed good antibacterial activity that was even better than the reference compound, ampicillin. S1 Table shows the zone of inhibition in millimeters (mm) as a measurement of antibacterial activity for all the *β-*lactam derivatives screened against the microbes. Calcium hydroxide, a common intracanal medicament in dentistry, showed no activity against *E*. *faecalis* at a concentration upto 200 μg/ml. So, calcium hydroxide was not used in further studies. Ampicillin showed no activity against resistant *E*. *faecalis* strains (RS1-5) at concentrations upto 100 μg/ml. So ampicillin was not used in further studies against resistant *E*. *faecalis* strains (RS1-5).

The MIC of *β-*lactam compounds were determined against *E*. *faecalis* (ATCC 29212), *S*. *aureus*, *Streptococcus* sp, and five resistant *E*. *faecalis* strains (RS1-5) by spectrophotometric method. The MIC of *β-*lactam compounds has been summarized in Table 1. Compound 3 showed MIC of 25 μg/ml against *E*. *faecalis*, resistant *E*. *faecalis* (RS1) and *Streptococcus* sp. It was compared with the reference compound, ampicillin (6 μg/ml). Based on the MIC values, three compounds namely, 3, 6a and 7 were chosen and considered for further studies.

**Table 1:** Minimum Inhibitory Concentration (μg/ml) of *β*-lactams against pathogens in root canal infection.

S.No	Compound name/ organisms	*E*. *faecalis* (ATCC)	*E*. *faecalis* (RS1)	*E*. *faecalis* (RS2)	*E*. *faecalis* (RS3)	*E*. *faecalis* (RS4)	*E*. *faecalis* (RS5)	*Streptococcus* sp	*S*. *aureus*
1	2a	50	100	-	-	100	100	50	100
2	3	25	25	50	100	50	100	25	50
3	6a	25	50	100	100	100	100	25	25
4	7	25	50	100	50	50	50	50	25
5	9	50	100	100	-	50	-	50	-
6	11a	-	-	100	100	-	100	-	-
7	11b	50	100	100	-	50	100	50	50
8	12	50	100	100	100	100	100	50	50
9	13a	50	100	100	-	50	-	50	-
10	13b	50	100	100	100	-	100	50	50
11	14	25	50	-	100	50	-	50	50
12	16a	-	-	-	-	100	100	-	25
13	18a	50	100	-	-	-	-	50	50
14	18b	50	100	100	100	50	-	100	50
15	19	100	150	100	100	100	100	100	-
RC	Amp	6	-	-	-	-	-	12.5	12.5

-, no activity, Amp- Ampicillin, RS (1–5)—Resistant strains isolated from Root Canal Treatment failure cases.

#### Time kill assay

**Fig 3:**
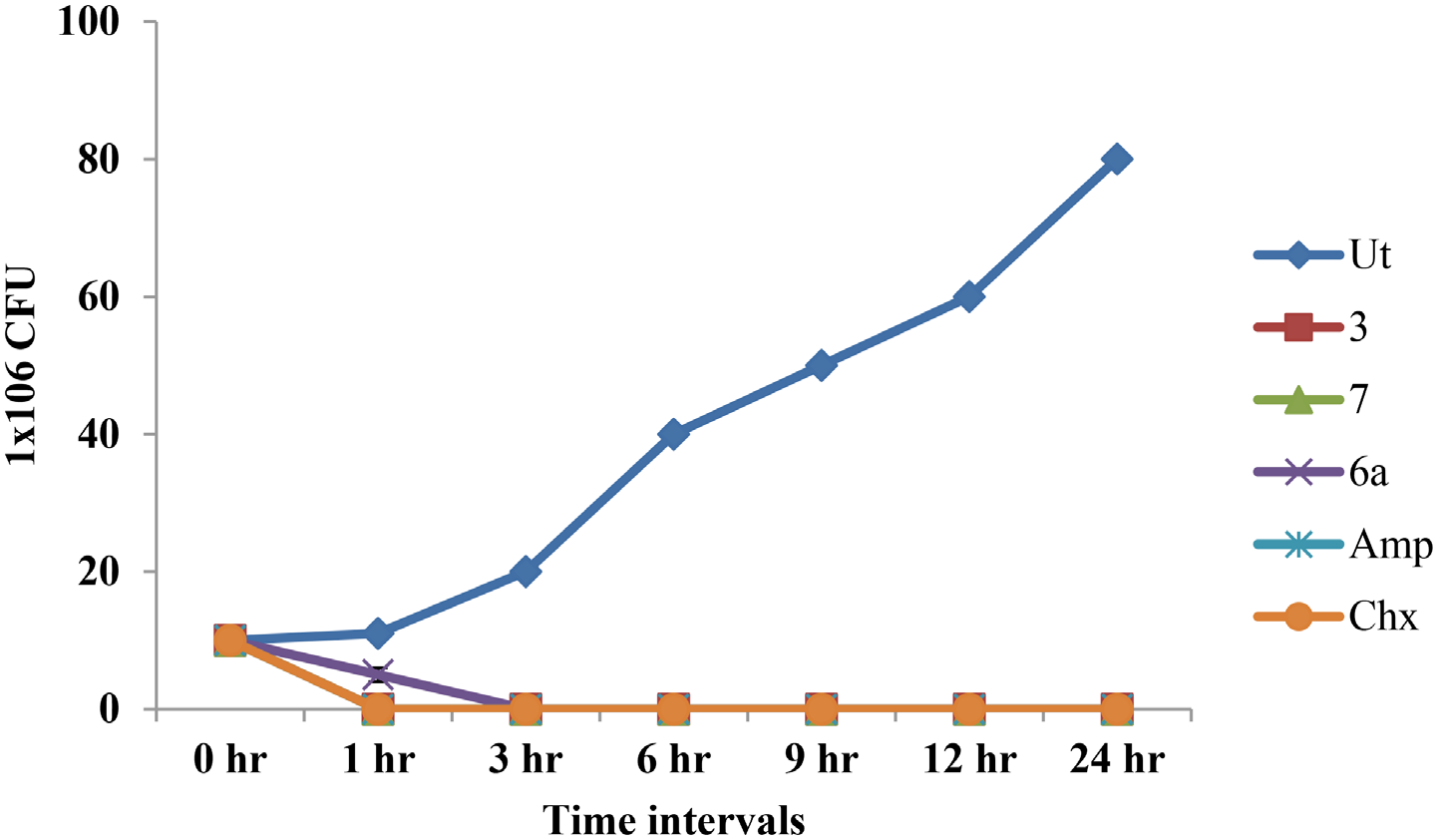
Time kill assay for *E*. *faecalis*. Time kill assay confirmed growth inhibitory effect of *β-*lactam compounds (3, 7 and 6a) against *E*. *faecalis*. Samples were collected at the indicated times and were evaluated for CFUs. Time kill kinetics of *E*. *faecalis* by compound 3 (25 μg/ml) represent dead cells within 1 h. Note: Ut- untreated, Amp- Ampicillin.

In the time kill assay, the growth inhibitory effect of *β-*lactam compounds 3, 6a and 7 was calculated against *E*. *faecalis*. The CFUs of *E*. *faecalis* cells were reduced after treatment with *β-*lactam compounds at concentrations equal to their MICs. No viable bacterial cells were observed after treatment of *E*. *faecalis* with compounds 3 and 7 for 1 h; whereas for compound 6a, no viable cells were present after 3 h treatment. Killing kinetics of *β-*lactam compounds was compared with ampicillin and it was observed that ampicillin and *β-*lactam derivatives exhibited similar killing kinetics (Fig 3). Based on time kill assay, compounds 3 and 7 were considered for further studies.

#### Antibacterial efficacy of *β*-lactam in e*x vivo* dentine model

**Fig 4:**
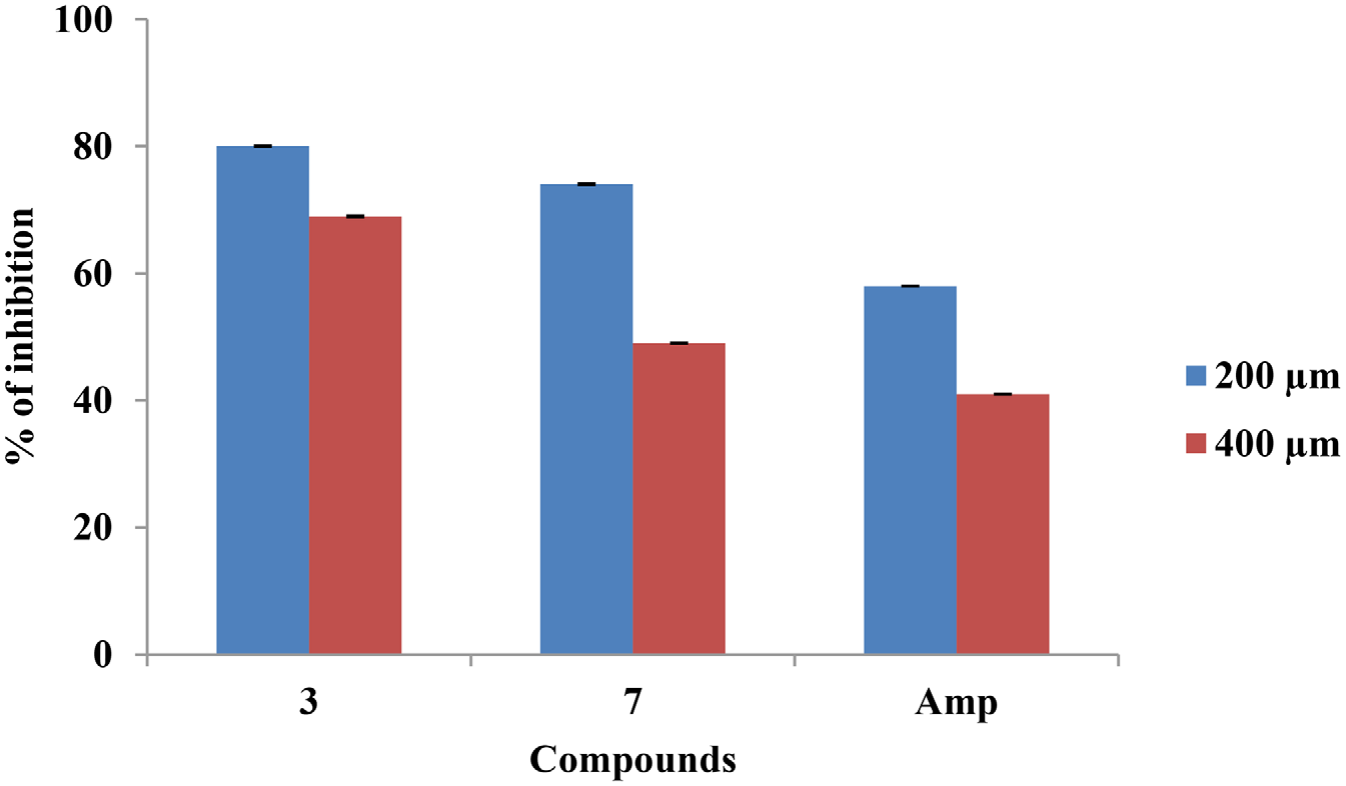
Efficacy of *β*-lactam compounds in *ex vivo* dentine tubule infection model. Tooth samples were infected with *E*. *faecalis* (1.5x10^5^) cells and were treated with *β-*lactam compounds (3 and 7) at their MICs. After incubation, dental chips containing resident microbes were harvested at two depths (200 and 400 μm). The mean value of culture O.D was obtained. The percentage of reduction was calculated. Non-parametric test was done for comparisons of significance for test versus control (*p*< 0.05). Note: Amp- Ampicillin.

The antibacterial activity of *β-*lactam compounds 3 and 7 was tested against *E*. *faecalis* in dentine model, which mimics the *ex vivo* condition of root canal infection. In the dentine model, the efficacy of *β-*lactam compound to eliminate microbes was studied at two different depths of 200 and 400 μm. The bacterial loads in treated samples were quantified by spectrophotometric method and CFU count. The results are presented in Table 2 and Fig 4. After a 24 h treatment, *β-*lactam compounds showed good activity in eliminating bacteria at both 200 and 400 μm depths. Compound 3 showed 80% and 69% of bacterial reduction at 200 and 400 μm respectively and compound 7 showed 74% and 49% of reduction at 200 and 400 μm, whereas for ampicillin the reduction observed was only 58% and 41% at 200 and 400 μm respectively. The efficacy data of the present study suggested that the compounds effectively penetrated the dentine tubules and inhibited the growth of *E*. *faecalis* even up to depth level of 400 μm and had better activity than ampicillin.

**Table 2:** Mean log CFU for different intracanal medicaments at 200 μm and 400 μm in *ex vivo* dentine model.

S. No	Test agent	200 μm		400 μm		*p* value
		Mean Log CFU count ±SD	Decrease in log CFU count vs culture control	Mean Log CFU count ±SD	Decrease in log CFU count vs culture control	
1	Untreated	11.1802 ± 0.043		10.025±0.039		
2	3	5.1769 ± 0.069	6.00332	5.653 ±0.189	4.37206	0.0001
3	7	7.5588±0.058	3.62142	8.402 ±0.241	1.6232	0.0001
4	Ampicillin	6.9832± 0.200	4.19705	6.890 ±0.192	3.13457	0.0001

#### *In vivo* antibacterial efficacy of *β*-lactam compound

**Fig 5:**
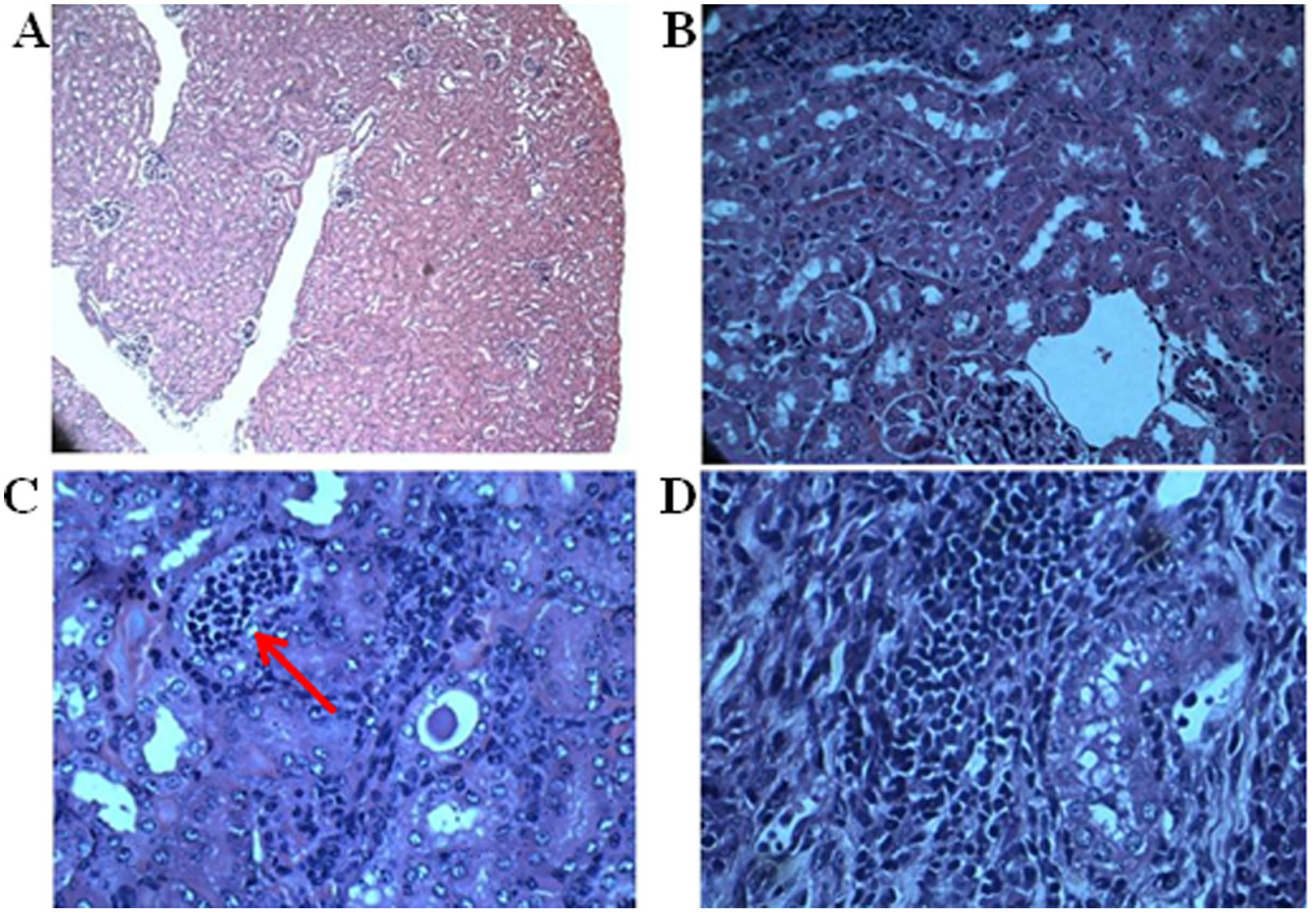
*In vivo* antibacterial efficacy of *β*-lactam compound. To determine the *in vivo* efficacy of compound 3, Balb/C mice were inoculated with *E*. *faecalis* and compound 3, Ampicillin and saline was administered once daily for three days. After 120 h, mice were killed and organs were isolated and histopathological examinations were conducted. Histopathological observation of *E*. *faecalis* infected mice kidney sections shows A) Kidney section from healthy uninfected Balb/C mice stained with haematoxylin- eosin. B) Moderate degree of tubular degeneration in 120 h after infection with *E*. *faecalis* C) Inflammatory cells in the interstitum along with the hyaline cast in kidney cells of high dose (50 mg/kg) of *β-*lactam treated group D) Interstital infiltration with inflammatory cells in ampicillin treated group.

*In vivo* antibacterial activity of *β*-lactam compound 3 was tested in an intravenous infection disease model using female Balb/C mice. Antibacterial efficacy was determined by counting the number of bacterial colonies in each organ. After 4 h of subcutaneous injection of low and high doses of *β*-lactam, no animals died in the treated groups but two mice died in the culture control group after 72 h of *E*. *faecalis* infection. Bacterial loads from blood, heart, lungs, brain, kidney and spleen were counted and calculated. The authors did not observe any growth in the blood, heart, lung and brain after 120 h time period in both treated as well as untreated groups; but the kidney showed higher bacterial load after 120 h of infection with *E*. *faecalis* and mice treated with low and high doses (25 and 50 mg/kg) of *β*-lactam compound 3 showed significant bacterial load reduction in the kidney (Table 3). The mean log CFU determined in kidney showed a 3log reduction in the lower dose of compound 3 and a 5log reduction in the higher dose of compound 3. Statistical analysis using Mann Whitney test showed that the log CFU reduction was significant with a *p* value of 0.036. Mice treated with ampicillin (25 mg/kg) showed less than 1log reduction of CFU in kidney, whereas mice treated with 0.1% DMSO did not show any CFU reduction in kidney. Histopathological lesions were observed in all the kidney sections (Fig 5). Inflammatory cells in the interstitium along with the hyaline casts were observed in kidney cells after infection with *E*. *faecalis*. Microscopic examination of kidney cells revealed moderate degree of tubular degeneration. Lesions were not seen in normal mice. Consistent histopathological lesions were observed in all the infected mice.

**Table 3:** *In vivo* anti bacterial efficacy of *β-*lactam in *E*. *faecalis* infected mice.

S. No	Antibacterial agents	Dose (mg/kg)	Mean log CFU count(Kidney) ±SD	Decrease in log CFU count vs culture control	*p* value
1	Culture control	-	8.2134± 0.22588		
2	Low dose *β-*lactam	25	5.1181 ± 1.08723	-3.0953	0.036
3	High dose *β-*lactam	50	3.1592 ± 0.96102	-5.0842	0.036
4	Ampicillin	25	7.5255 ± 1.60899	-0.6879	0.786
5	0.1% DMSO	-	8.7491 ± 0.39501	0.5357	0.143

Five mice in each group received three subcutaneous doses of *β-*lactams (25 and 50 mg/kg) and ampicillin (25 mg/kg) after 4 h of intravenous inoculation of *E*. *faecalis*. 120 h after infection and daily treatment with dose indicated, the kidney were harvested and CFU were measured in kidney. Non-parametric test was done for comparisons of significance for test versus control (*p*< 0.05).

#### Covalent docking analysis

**Fig 6:**
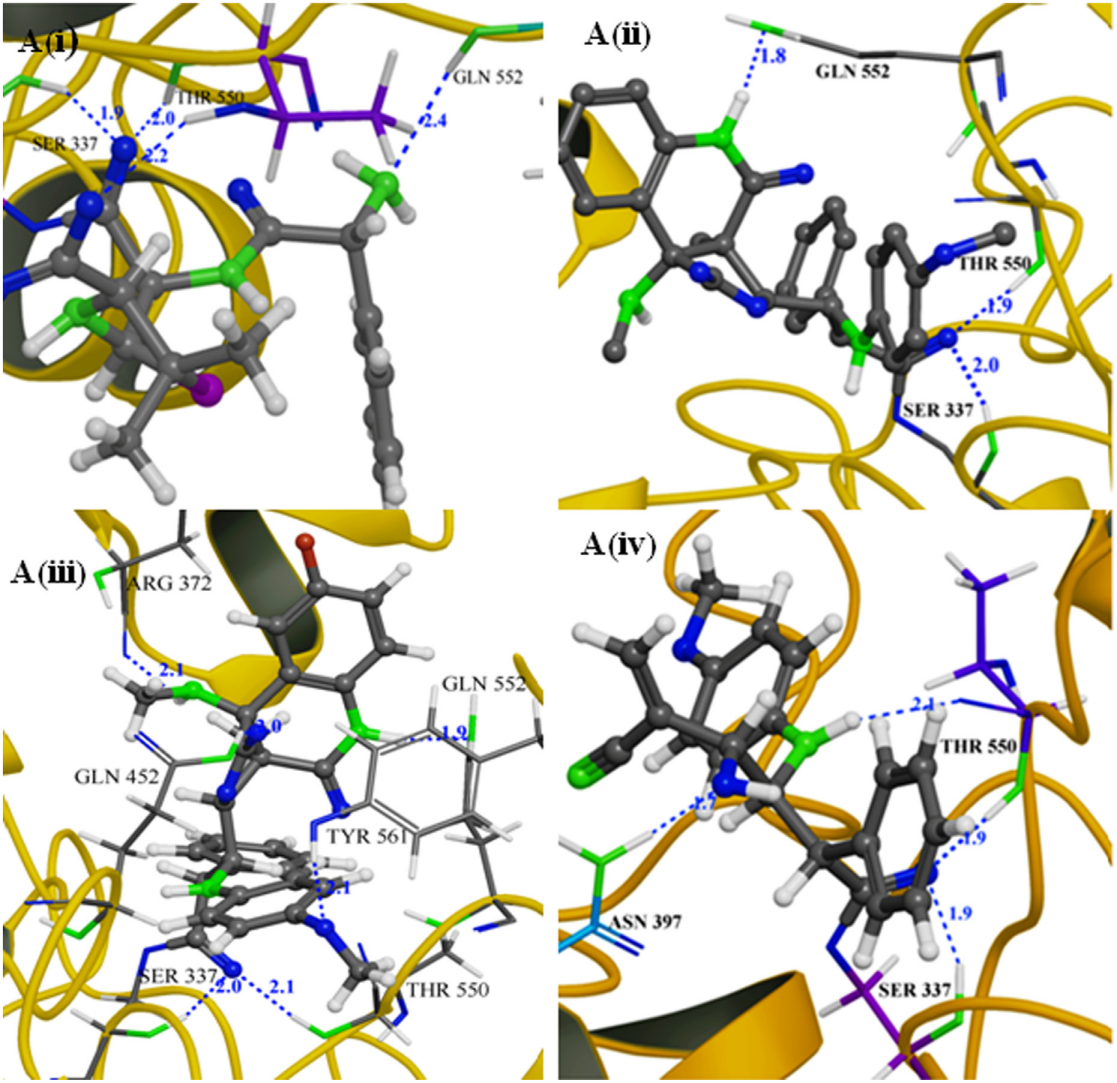
*In silico* studies: Binding posses of A(i) Ampicillin A(ii) Compound 7 A(iii) Compound 6a and A(iv) Compound 3 to the active site pocket of PBP (PDB ID: 2Z2M).

Covalent docking was performed to understand the antibacterial activity of *β-*lactam compounds. In docking studies, ligand was docked into the active site of penicillin binding protein (PBP) which provide covalent and non-covalent interactions profiles (Fig 6 (i-iv)). The CovDock method was examined by redocking with the experimental ligand into the binding site of PBP (PDBID: 2Z2M). Ligand RMSD values were calculated between the crystal structure and lowest energy of docked ligand pose which is found to be of 0.7 Å. The validation of covalent docking method result was found to be significant. The docking result of the reference compound Ampicillin provided the binding affinity, docking score, and binding energy of—7.19, -7.64 and -67.96 kcal/Mol respectively and it also shows interactions with three residues namely Ser337, Thr550, and Gln552. The amide group of four membered ring in the *β*-lactam synthesized compound was found to be interacting with Ser337. In this reaction, *β*-lactam was found to act as an electrophile whereas the covalent reactive residue of Ser337 acts as a nucleophile. 2D representations of three best *β*-lactam compounds are interacting with PBP shown in S4 Fig. The hydroxyphenyl group substituted at para N1 position of lactam ring is positioned into the hydrophobic pocket which consists of Phe450, Tyr561, Ala551, Trp374, Tyr568, and Ile371 residues. Covalent docking affinity for the compounds 7, 6a and 3 are -8.57, -8.39, and -8.28 kcal/Mol respectively. The predicted inhibitory potency of synthesized *β*-lactam is presented in Table 4. The compounds 7, 6a and 3 were found to have strong hydrogen bonds interactions which resulted in higher binding affinity. The result reveals that these compounds are found to interact commonly with Ser337 and Thr550. Based on the significant docking score, it is affirmed that these three compounds have potent inhibitory activity against the PBP. To summarize, all the synthesized compounds had an overall high score and thereby demonstrated better inhibitory effect against the PBP. The compounds forming hydrogen bonds showed better interactions. Hence, the synthesized compounds 6a, 7 and 3 showed better binding ability than ampicillin based on docking score.

**Table 4:** Docking score of *β*-lactam derivatives.

S. No	Compounds	Cdock Affinity (kcal/mol)	Glide Score (kcal/mol)	MMGBSA dG Bind (kcal/mol)	H-bond Interaction residues
1.	Ampicillin	-7.19	-7.64	-67.96	Ser 337, Thr 550, Gln 552
2.	6a	-8.57	-8.85	-84.46	Ser 337,Thr 550, Gln 552
3.	7	-8.39	-8.73	-69.08	Ser 337, Thr 550,Arg 372, Gln 452, Gln 552, Tyr 561
4.	3	-8.28	-8.66	-71.23	Ser 337,Thr 550,Asn 397
5.	2a	-7.89	-7.77	-63.24	Lyn340, Gln 452, Ser 337, Asn 397
6.	12	-7.86	-7.99	-88.63	Ser 337,Thr 550
7.	9	-7.82	-8.55	-93.10	Ser 337, Thr 550, Gln 552
8.	13a	-7.81	-8.64	-86.20	Ser 337, Arg 372, Gln 452
9.	18b	-7.65	-8.25	-91.79	Ser 337, Thr 550
10.	13b	-7.63	-7.95	-76.53	Ser 337, Gln 552
11.	16a	-7.60	-7.87	-62.35	Ser 337, Arg 372, Gln 452
12.	18a	-7.48	-7.79	-80.81	Ser 337, Thr 397
13.	11b	-7.37	-7.41	-44.86	Ser 337, Gln 550
14.	14	-7.36	-8.83	-102.76	Ser 337, Ser 548
15.	19	-7.24	-7.90	-60.01	Ser 337, Gln 552
16.	11a	-7.22	-7.47	-77.39	Ser 337, Thr550

#### Bacterial viability assay in *ex vivo* dentine model

**Fig 7:**
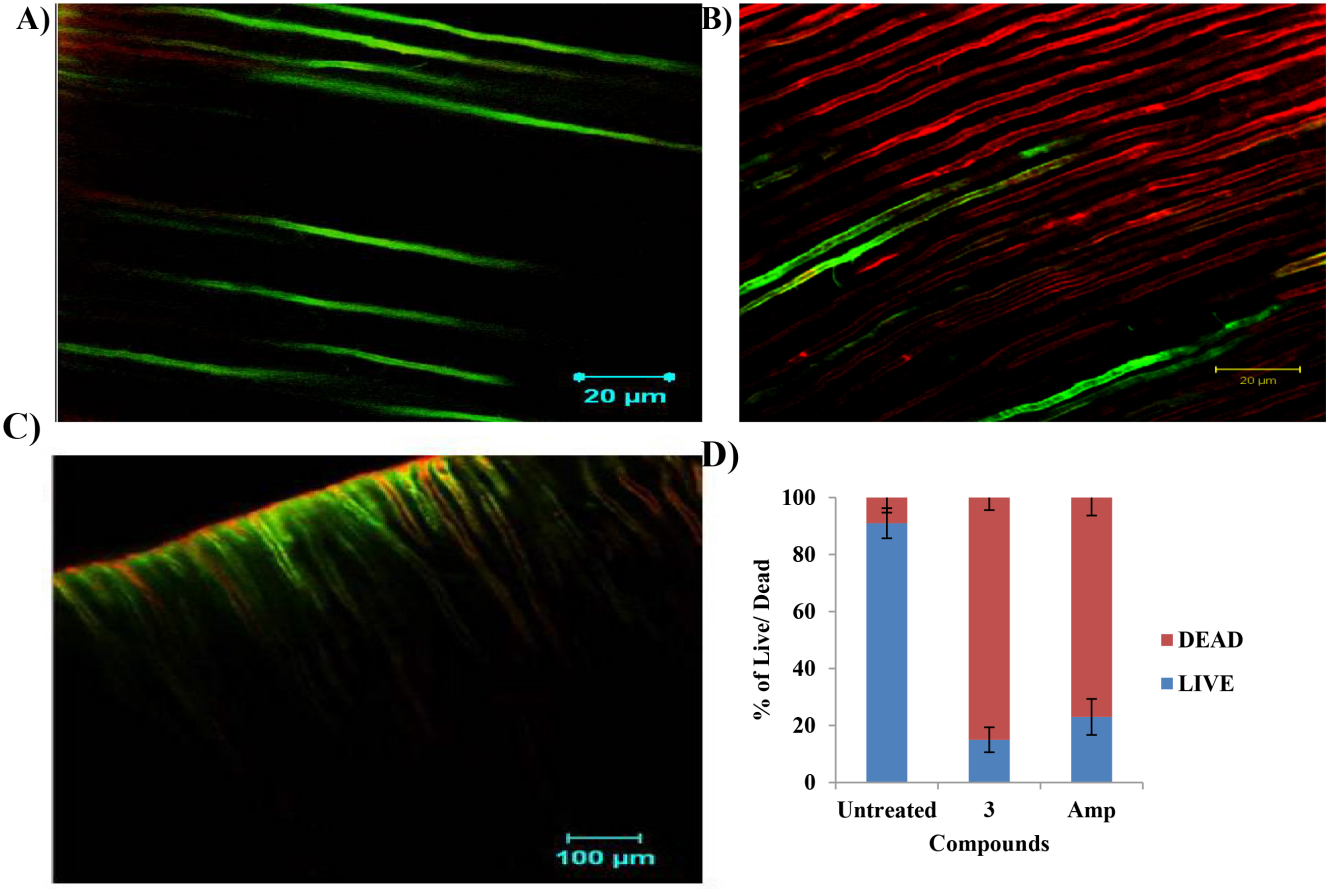
Bacterial live/ dead assay: Tooth samples were infected with *E*. *faecalis* (1.5x10^5^) cells for 21 days and were treated with 25 μg/ml of *β-*lactam compound 3 (MIC). After incubation, the tooth samples were sliced and stained with fluorescein diacetate/ propidium iodide. A) CLSM image of untreated root canal showed green fluorescence indicating live bacteria B) Marked red fluorescence in the root canal treated with *β-*lactam compound 3 shows dead bacterial cells C) Root canal treated with ampicillin shows red fluorescence. Note: scale bar: A) 20 μm B and C) 100 μm. D) Graph depicting the percentage of bacterial viability after treatment with various agents. Nonparametric test was done for comparisons of significance for test versus control (*p*< 0.05). Note: Amp- Ampicillin.

The bactericidal action of *β*-lactam 3 against *E*. *faecalis* was visually observed in CLSM after 24 h of treatment. For this purpose, tooth samples were contaminated with *E*. *faecalis* for 21 days, treated with *β*-lactams and viability was determined by using fluorescein diacetate (FDA, emit green fluorescence in live cells) and propidium iodide (PI emits red fluorescence, enters cells that have disrupted cell membranes and shows enhanced fluorescence by binding to DNA). On the other hand, FDA is a non-fluorescent dye, which is actively taken up by live cells and is hydrolyzed by the enzyme in the live cells, which emit green fluorescence when excited. This dye combination provides visual evidence for the viability of bacterial cells in the root canal system. By differential staining of cells with FDA and PI, the authors computed the percentage of cells with compromised membranes to the total number of cells. Results showed that *β*-lactam compounds were able to permeate bacterial cell membranes at its MIC after incubation for 24 h. Fig 7A–7C shows FDA and PI fluorescence levels in the *β-*lactam treated cells in the root canals. However, root canals without *β*-lactam treatment showed green fluorescence and minimal red fluorescence. As these live cells had intact membranes, PI was not able to penetrate the cells. Seven fields were examined and values were obtained for calculating the live/dead ratio. The results of the present study indicated that compound 3 treated root canals showed 80.65% of dead and 19.35% of live cells; whereas for ampicillin it was 63.14% and 36.82% of dead and live cells respectively. The results suggested that compound 3 showed efficient activity against *E*. *faecalis* in the root canal system (Fig 7D).

#### Morphological changes by scanning electron microscopy

**Fig 8:**
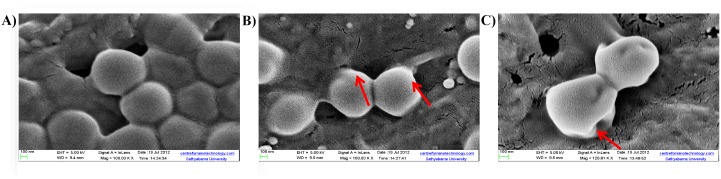
Scanning Electron Microscopy. Scanning Electron Microscope to show membrane damage after treatment with various agents A) untreated cell with intact surfaces B) *β-*lactam compound 3 induced cell damage in the form of cell swelling (arrows indicate areas of damage) C) damage to the bacterial cell after treatment with ampicillin. Note: Size bars: 100 nm.

Morphological changes were seen in *E*. *faecalis* cells using SEM to understand the mechanism of action of synthesized *β-*lactam compound against *E*. *faecalis*, (Fig 8). Cells treated with *β-*lactam compound 3 (25 μg/ml) for 1 h, showed cell swelling and altered morphology of the cell as documented in the SEM image; whereas cells treated with ampicillin showed cell damage in the form of cell shrinkage.

#### Generation of reactive oxygen species

**Fig 9:**
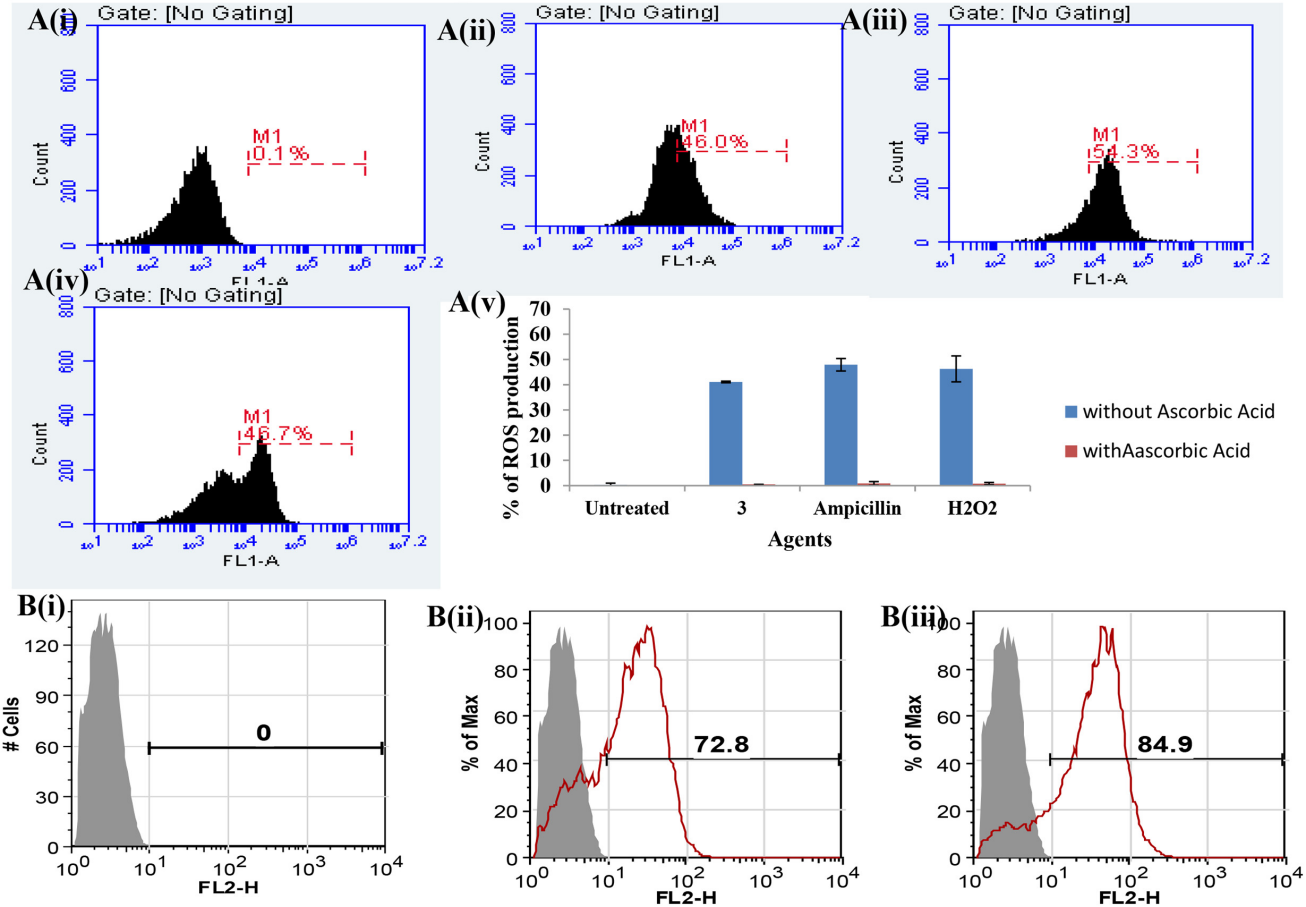
Mechanism of action of *β*-lactam compound 3. A) FACS based measurement of ROS Production. A(i) Untreated cells showed no fluorescence indicating no ROS production. A(ii) Cells showed fluorescence by compound 3 was monitored by incubation of compound 3 (25 μg/ml) with *E*. *faecalis* cells for 1 h. Green fluorescence indicating generation of ROS production after treatment with compound 3. A(iii) Cells showed fluorescence indicating generation of ROS production after treatment with ampicillin. A(iv) Cells showed fluorescence after treatment with hydrogen peroxide (H_2_O_2_) A(v) Graph indicating the percentage of ROS production after treatment with respective agents. The mean and standard deviations from six triplicates were plotted. B) Flow cytometry analysis to show membrane permeabilization of *E*. *faecalis* cells in PI uptake assay. *E*. *faecalis* (10^6^ cells) cells were incubated with respective agents for 45 min. B(i) Untreated cells showed no fluorescence indicating no membrane damage B(ii) Cells incubated with *β*-lactam compound 3 (25 μg/ml) showed increased PI uptake as compared to untreated indicating membrane damage and B(iii) Cells incubated with ampicillin showed increased PI uptake.

The formation of ROS has been suggested to be one of the bactericidal mechanisms of action of *β*-lactam compound 3 [38]. In order to determine the mechanism of killing of *E*. *faecalis* was mediated by ROS, we treated the cells with compound 3 at 25 μg/ml (MIC) for 1 h in the presence and absence of known anti oxidant, ascorbic acid at 100 μg/ml. ROS generation was measured by an increase in fluorescence intensity. A green fluorescence, resulting from oxidation of dye DCFH-DA was observed indicating the presence of ROS by compound 3. No fluorescence was observed when the cells were incubated with compound 3 in the presence of the antioxidant ascorbic acid (Fig 9A (i-v)); whereas untreated cells showed no fluorescence.

#### Membrane permeabilization assay

FACS based membrane permeabilization of *β*-lactam compound 3 on *E*. *faecalis* cells was studied using the DNA binding dye, propidium iodide. *E*. *faecalis* cells showed membrane damage when treated with *β*-lactam compound at its MIC for 45 min (Fig 9B (i-iii)). The membrane damaged cells only allowed the PI to enter into the cells, whereas untreated cells showed no fluorescence.

#### Qualitative and quantitative biofilm assay

**Fig 10:**
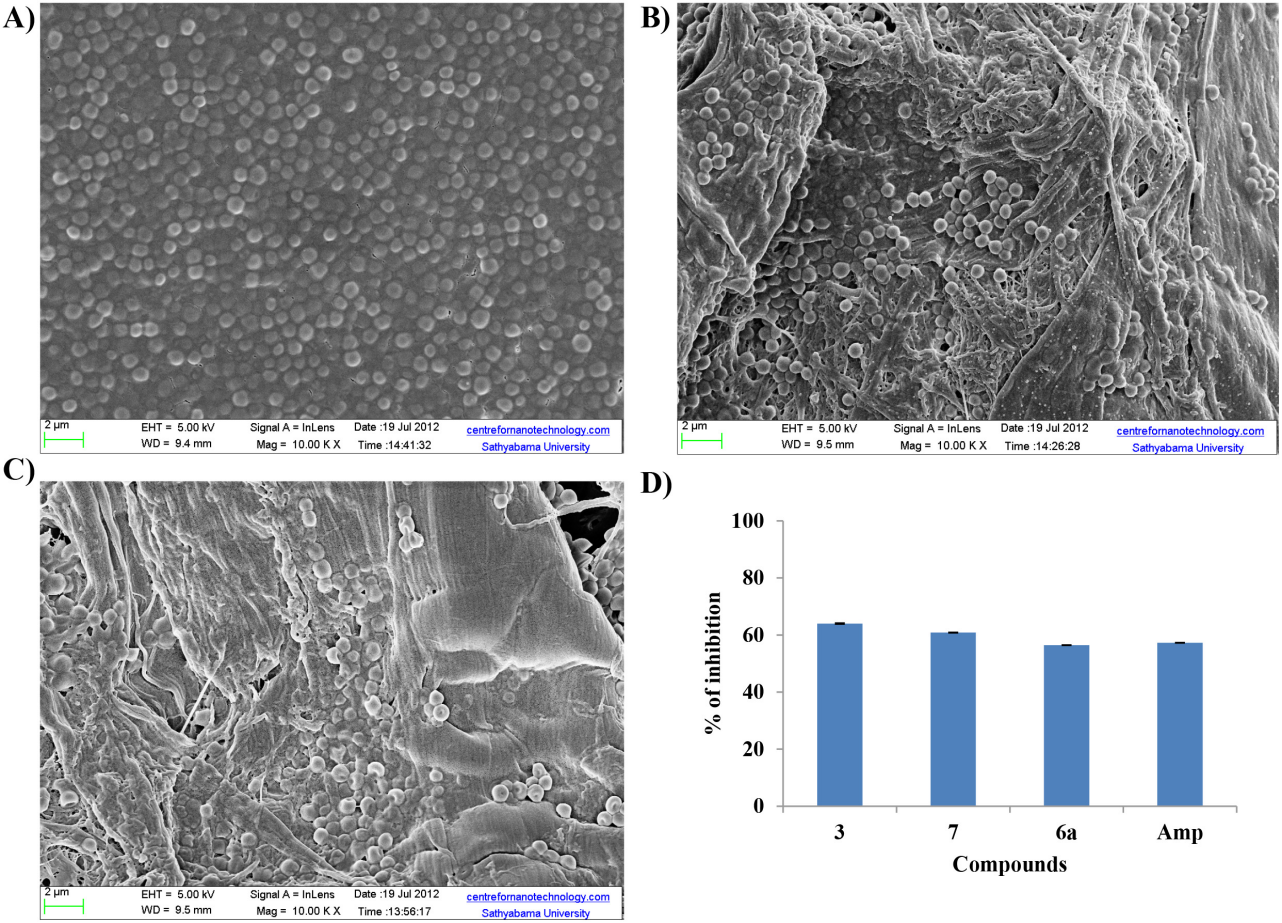
Biofilm assay. Qualitative SEM study to show inhibition of biofilm formation. Images show reduction of biofilm after 24 h treatment with agents A) Large number of viable adherent bacterial cells on the matrix (control without treatment) B) bacterial reduction after treatment with *β-*lactam compound 3 (25 μg/ml) C) bacterial reductions after treatment with ampicillin. Notes: scale bar: 2 nm. D) Quantitative spectrophotometric measurement to show inhibition of biofilm formation. Graph indicates percentage of biofilm reduction after 24 h treatment with respective agent. Nonparametric test was done for comparisons of significance for test versus control (*p*< 0.05). Note: Amp- Ampicillin.

The inhibitory effect of *β*-lactam compounds on biofilm formation was carried out on cellulose matrices. In this qualitative method, biofilms of *E*. *faecalis* treated with *β*-lactam compounds and controls were analyzed under SEM. *β*-lactam compound 3 significantly reduced the number of viable bacterial cells on matrix and inhibited biofilm formation (Fig 10A–10C) and the reduction was comparable to the ampicillin. The activity of new *β*-lactam on biofilm formation was studied quantitatively by the 96 well microtitre plate method. Our results showed 65% biofilm reduction by compound 3, compound 7 showed 60% reduction and compound 6a showed 56% reduction; whereas for ampicillin the reduction observed was only 51%. The results are represented in Fig 10D.

#### Hemolytic assay

Hemolytic assay was used to ascertain the acute toxicity of the *β*-lactam compounds on RBCs. None of the *β-*lactam compounds were hemolytic at the concentration equal to their MICs. The percentage of hemolysis was found to be 0.67%, 0.9% and 7.5% respectively for compounds 3, 6a and 7 respectively, whereas it was 11.6% for ampicillin. Based on these results the *β*-lactam compounds were found to be non hemolytic. The results are presented in S2 Table.

#### Mammalian cell cytotoxicity

**Fig 11:**
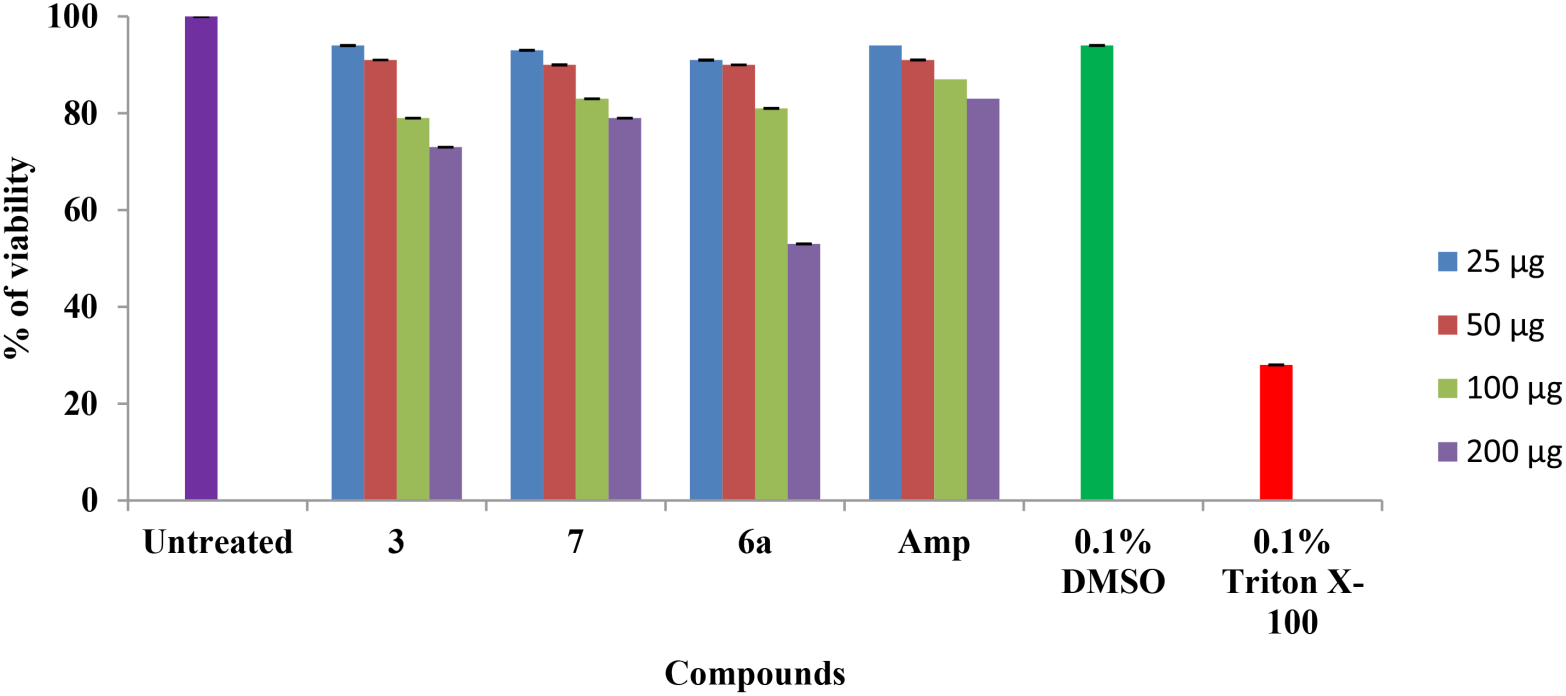
Mammalian cell cytotoxicity. MTT based cytotoxicity assay on NIH 3T3. The cells were treated with various concentrations of *β-*lactam compounds and incubated for 24 h. After incubation, the percentage of cell viability was calculated. Standard deviations from three observations were plotted. Note: Amp- Ampicillin.

The cytotoxicity of *β-*lactam compounds was tested on mammalian cell line (NIH 3T3 mouse fibroblast) by the MTT assay. Here, the cell viability was measured after treatment with various concentrations of the *β-*lactam compounds (Fig 11). After 24 h of incubation, none of the newly synthesized *β-*lactam compounds showed toxicity to NIH 3T3 cells. Compound 3, 6a and 7 showed 94%, 93% and 91% viability to NIH 3T3 cells with their respective MICs. However, we observed that at 8X MIC values, compound 3, 6a and 7 treated cells had 73%, 79% and 53% of viability respectively. Ampicillin showed 83% cell viability to NIH 3T3 at their 8X MIC. These results indicated that the toxicity was observed only at higher concentrations (8X MIC) and not at their respective MICs. Hence *β-*lactam compounds could serve as effective antibacterial drugs with a good safety profile for treating root canal infection.

#### *In vitro* genotoxicity assays

Ames test was done to study the mutagenic potential of *β*-lactam compounds in *Salmonella typhimurium* strains TA 98 and TA 100 without metabolic activation system. *β*-lactam compounds 3 and 7 incubated with *Salmonella typhimurium* strains did not form any colonies whereas mitomycin C and sodium azide treated strains formed revertant colonies. The colonies were counted and the values were also statistically significant between *β*-lactam compounds and sodium azide and mitomycin C treated groups (S3 Table). Genotoxic effect of *β*-lactam compounds (3 and 7) were studied *in vitro* on human peripheral blood lymphocytes using the chromosomal aberration and micronucleus assays. Both *β*-lactam compounds 3 and 7 did not show any chromosomal aberration or micronuclei formation at 10X MIC (250 μg /ml) (data not shown).

#### Wing spot assay

*In vivo* genotoxicity of *β*-lactam compound was determined by SMART in *Drosophila melanogaster*. In this experiment, 3^rd^ instar transheterozygous larvae (mwh+/ Flr^3^+) were used for the recessive genetic markers of mwh and flr^3^. Statistical analysis using Dunnett t (2-sided) test showed that, there was no statistically significant difference in genotoxicity of the untreated and *β*-lactam compound treated group (*p* > 0.922) as compared to the positive control EMS (*p* < 0.026). This shows that, *β*-lactam compound did not induce any genotoxic effect at the defined doses (S4 Table).

## Discussion

The oral cavity harbors maximum microorganisms in the body and among them bacterial population is predominant [39]. From the oral cavity, microorganisms may have access to the root canal through the crown or root following traumatic pulp exposure, dentinal tubules following carious invasion and blood or lymph [40]. Persistence of microbes in the root canal leads to treatment failure. Several studies have revealed that *E*. *faecalis* is the most frequent species in root canal treated teeth, with prevalence values reaching up to 90%. Calcium hydroxide is the only intracanal medicament currently used in endodontic infections. Several studies have reported that calcium hydroxide is not effective in eliminating *E*. *faecalis*, because it forms biofilms in root canals which contribute to resistance [2, 41, 42]. Apart from calcium hydroxide, antibiotic-containing preparations were also used in endodontic therapy as topical agents. However, the potential risk for bacterial resistance and drug hypersensitivity limits their usefulness in endodontic treatment [43, 44]. To overcome the limitation of the existing medicaments, the authors of the present study have described the antibacterial activity of *β*-lactam derived polycyclic fused pyrrolidine/pyrrolizidine derivatives synthesized by 1, 3-dipolar cycloaddition reaction against *E*. *faecalis*, as a future intracanal medicament.

In the present work, the authors screened fifteen *β*-lactam derived polycyclic fused pyrrolidine/pyrrolizidine derivatives against microbes involved in dental infection and showed activity as similar to reference compound ampicillin. The antibacterial efficacy of *β*-lactam compound against *E*. *faecalis* was evaluated by counting colony forming units (CFU/mg of dentin) in *ex vivo* dentine model, which mimicked the *in vivo* condition. Human premolar single-rooted tooth samples were contaminated with *E*. *faecalis* for 21 days to form mature biofilm in dentin. *β*-lactam compound was packed inside the root canal as medicament for 24 h and viable bacterial cells were quantified in two different depths (200 and 400 μm). The results showed 6log and 4log reduction in 200 and 400 μm due to good stability, solubility and diffusibility of *β*-lactam derivative, whereas previous results showed 5log reduction after treatment with calcium hydroxide in combination with 25% of chitosan solution at both depths [17].

To determine the antibacterial activity of *β*-lactam derivatives under *in vivo* conditions, the authors used an intravenous mice model, which is the most effective and accepted model to study the *E*. *faecalis* infection. Though our study was focused on root canal infections, at present there are no small animal models to study the efficacy of new agents in root canal infection. Hence, this intravenous model was used to show the antibacterial activity of *β*-lactam derivatives that resulted in bacterial reduction after treatment. The bacterial burden and organ histopathology of intravenously infected mice were observed for 120 h. In concurrence with earlier reports [18], the authors did not find any bacterial growth in brain, lung, heart and blood but a high bacterial burden was observed in kidneys. Treatment with *β*-lactam derivatives significantly reduced the bacterial growth in kidneys. The number of bacterial colonies present in kidney was correlated with its severity of histopathological lesions. The authors observed 5log bacterial reductions in high concentration (50 mg/kg) of *β*-lactam derivatives. This reduction was very significant in animal models when compared with the earlier reports, where ampicillin (50 mg/kg) was used in the same infectious model [45]. The reduction by compound 3 in intravenous model was observed to be better than daptomycin in intravenous model reported earlier [46]. *In vivo* studies undoubtedly showed that newly synthesized compound 3 had good efficacy and good stability in eradicating *E*. *faecalis*.

*β*-lactam derivatives are known for their mechanism of action by inhibiting cell wall synthesis [47]. It is known that, bacterial cell wall composed of peptidoglycan determines the cell wall rigidity, cell shape and prevent cells from osmotic shock [48]. The biosynthesis of bacterial cell wall peptidoglycan is mediated by the penicillin binding protein (PBP). PBPs are membrane bound enzymes involved in the final steps of the bacterial cell wall synthesis on the periplasmic side of the membrane [49]. To find out the mechanism of action of *β*-lactam derivatives, *in silico* docking was performed against PBP, which showed that the derivatives were bound to SER337 of the protein and blocked the further binding of peptidoglycan for cross linking; thus acting as potential ligands. Considering the binding mode of all the compounds and positioning of the ligand in the active site, it is understood that the ligands play a major role in cross linking of the peptidoglycan synthesis in the cell wall. The docking studies of these *β*-lactam derivatives clearly stated the importance of the mode of action through interactions between *β*-lactam derivatives and the targeted protein. *β*-lactams antibiotics have been shown to interfere with specific steps in cell wall biosynthesis by binding to the PBP, resulting in the formation of weakened cell wall and osmotically unstable cells susceptible for lysis. Reports have shown that treatment with a cell wall synthesis inhibitor result in changes in cell shape and size, induction of cell stress responses and eventually cell lysis [50]. Our SEM analysis indicating damage of *E*. *faecalis* treated cells is in alignment with published studies. Cell damage was further corroborated by ROS generation; since, during the cell wall synthesis lipid II is the precursor for peptidoglycan biosynthesis and substrate for PBP [48] and ROS production damages macromolecules like protein, membrane lipid and DNA [51]. Thus our results show that the *β*-lactam compound 3 may have two modes of action (i) direct interaction with PBP, based on our *in silico* results which show the evidence for the compound 3 interaction with PBP and SEM result shows altered *E*. *faecalis* cell morphology after treatment with compound 3. Another recent report suggest that, *β*-lactams compounds inhibit cell wall synthesis through the bulge mediated mechanisms [52] (ii) Generation of ROS leads to cell membrane damage evidenced by an increase in PI uptake (72.5%) after treatment with compound indicating permeabilized bacterial cell membrane. The PI uptake result correlated well with an earlier study, wherein cell membrane damage in *E*. *coli* after treatment with cationic antimicrobial peptide at 90 min showed 95% of PI uptake [28].

The ultimate aim of the present study was to develop these compounds for human applications. In this regard, as compound 3 showed good antibacterial efficacy, a battery of *in vitro* and *in vivo* genotoxicity studies were performed to ascertain the safety profile. Genotoxicity studies showed that the compound was not toxic to human erythrocytes, lymphocytes, mammalian fibroblast cell lines and not mutagenic in bacterial reverse mutation assay. The *in vivo* genotoxicity studies on *Drosophila* model also showed that compound 3 was not toxic. This safety profile gave the confidence that compound 3 would prove to be an useful antibacterial agent.

### Conclusion

A total of fifteen *β-*lactam compounds were synthesized by 1, 3-cycloaddition methodology and were tested for anti bacterial activity to *E*. *faecalis*, *Streptococcus*, *S*. *aureus* and five resistant *E*. *faecalis* implicated in root canal infections. Among the fifteen compounds, compound 3 had an MIC of 25 μg/ml and was found to be efficacious against *E*. *faecalis* as an intracanal medicament in the dentinal tubule model. The *in vivo* mice infection model showed that compound 3 treated mice had reduced bacterial load in kidney as compared to the ampicillin treated group. The compound caused membrane damage to *E*. *faecalis* cells, inhibited biofilm formation and a CLSM study showed that it eradicated maximum of 80.65% of bacteria in the dentinal tubules. The bioactive *β-*lactam compound 3 was found to be nontoxic to mammalian fibroblast, human RBCs and lymphocytes and was also found to be nonmutagenic. The compound has potential clinical applications.

## Supporting Information

S1 Fig X-ray crystal structure of compound 6a. (TIF)

S2 Fig X-ray crystal structure of compound 18a. (TIF)

S3 Fig X-ray crystal structure of compound 19. (TIF)

S4 Fig 2D representations of *β*-lactam compounds. (TIF)

S1 File Experimental and spectral details of β- lactam compounds. (DOCX)

S1 Table Zone of inhibition of *β*-lactams. (DOCX)

S2 Table Hemolytic assay. (DOCX)

S3 Table Ames test. (DOCX)

S4 Table Wing spot assay. (DOCX)
