# Supplementary figures and images for: Bioactivity Studies of β-Lactam Derived Polycyclic Fused Pyrroli-Dine/Pyrrolizidine Derivatives in Dentistry: In Vitro, In Vivo and In Silico Studies
Source: PLoS One. 2015 Jul 17;10(7):e0131433. doi: 10.1371/journal.pone.0131433 (PMC4505899; doi:10.1371/journal.pone.0131433)

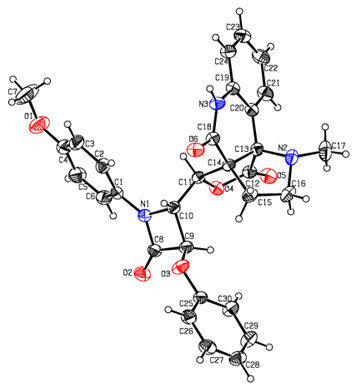

Supplement: S1 Fig — (TIF) [file pone.0131433.s001.tif]

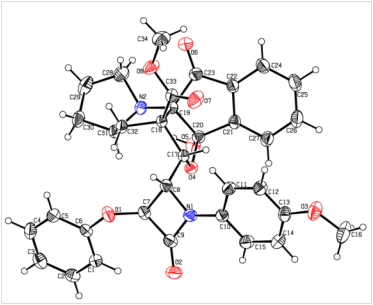

Supplement: S2 Fig — (TIF) [file pone.0131433.s002.tif]

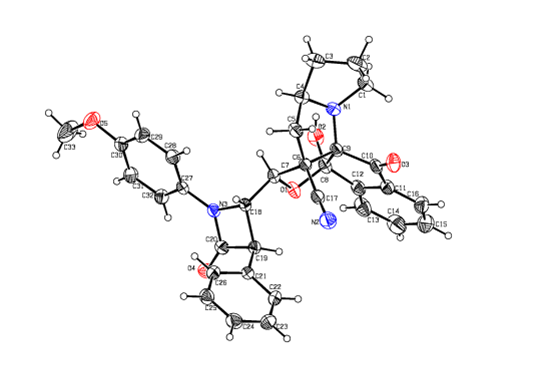

Supplement: S3 Fig — (TIF) [file pone.0131433.s003.tif]

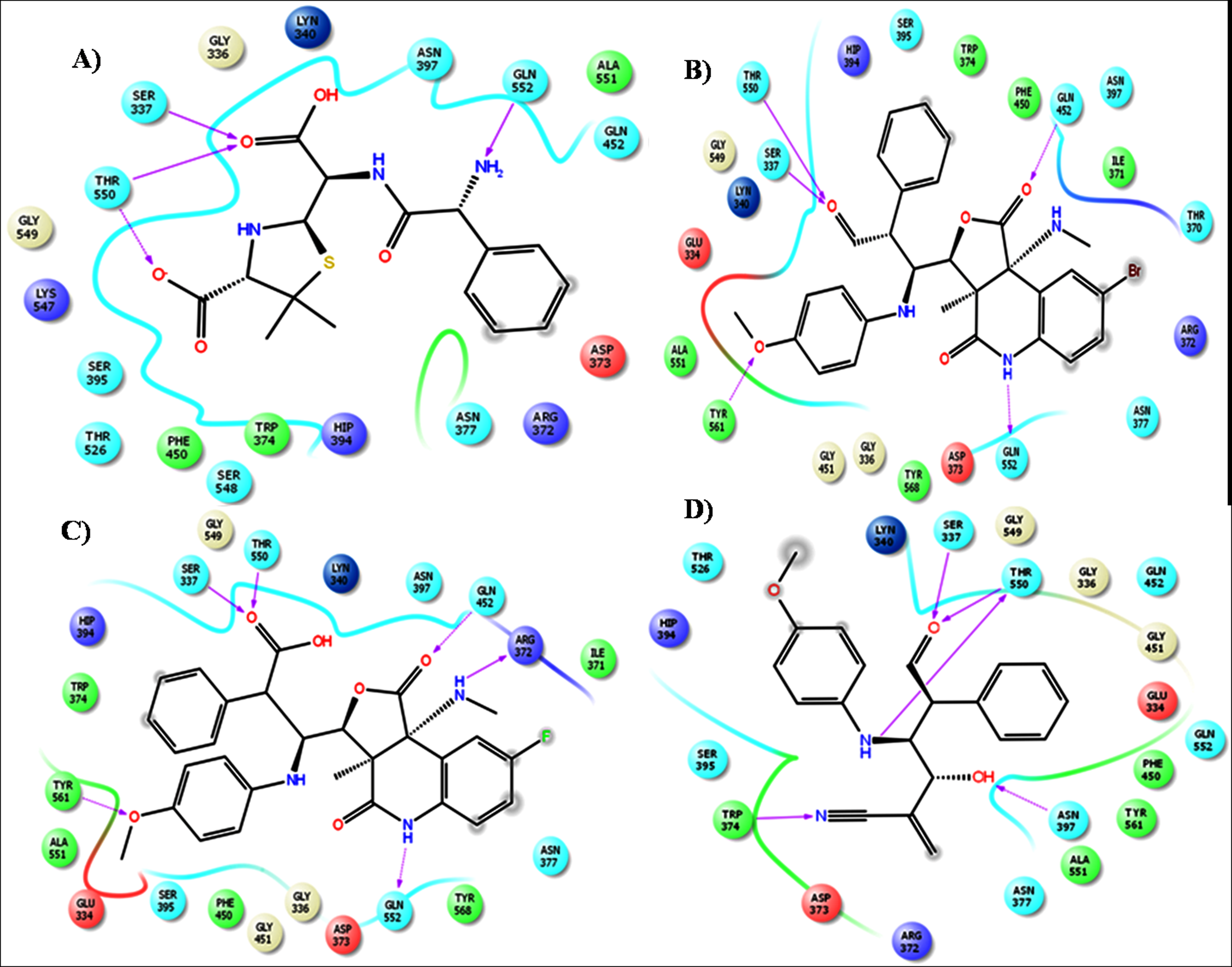

Supplement: S4 Fig — (TIF) [file pone.0131433.s004.tif]
