# Supplementary material for: Bioactivity Studies of β-Lactam Derived Polycyclic Fused Pyrroli-Dine/Pyrrolizidine Derivatives in Dentistry: In Vitro, In Vivo and In Silico Studies
Source: PLoS One. 2015 Jul 17;10(7):e0131433. doi: 10.1371/journal.pone.0131433 (PMC4505899; doi:10.1371/journal.pone.0131433)
Supplement: S1 File — (DOCX) [file pone.0131433.s005.docx]

**Supporting Information**

**Experimental Section**

**General Methods:** All melting points were uncorrected. The progression of all the reaction was monitored by TLC using hexanes/ethyl acetate mixture as eluent. Column chromatography was carried out on Silica gel by using increasing polarity. ^1^H, ^13^C and DEPT-135 spectra were recorded in CDCl_3_ using TMS as an internal standard on a Bruker 300 MHz spectrometer at room temperature. Chemical shift values are quoted in parts per million (ppm) and coupling constants (J) are quoted in Hertz (Hz). Mass spectra were recorded on JEOL GC mate mass spectrometer. The X-ray diffraction measurements were carried out at 298 K on a Bruker (2008) SMART APEX 2 area detector diffractometer.

**General procedure for the synthesis of Baylis –Hillman adducts (2a,b, 3)**

A mixture of 4-oxoazetidine-2-carbaldehyde 1a, b (25 mmol, 2.653 g), methyl acrylate (37.5 mmol, 3.228 g) and DABCO (3.75 mmol, 0.420 g) was kept at room temperature for 12-15 days. After completion of the reaction as evidenced by TLC analysis, the reaction mixture was diluted with ethylacetate (20 mL) and washed successively with 2N HCl solution, water and aqueous NaHCO_3_ solution. Organic layer was dried over anhydrous Na_2_SO_4_. Solvent was evaporated under reduced pressure and residue was purified by column chromatography using hexane: EtOAc (8:2) as eluent to afford Baylis –Hillman adducts (2a,b, 3) in good yield.

**Compound 2a:** White solid (75 %), Mp: 96-98 ºC; IR (KBr): 3472, 1744, 1705 cm^-1^; ^1^H NMR (300 MHz, CDCl_3_): δ 2.61 (br s, 1H, -OH), 3.77 (s, 3H), 3.79 (s, 3H), 4.33-4.34 (t, J = 2.1, 1.8 Hz, 1H), 4.49-4.50 (d, J = 2.1 Hz, 1H), 5.23 (s, 1H), 6.21 (s, 1H), 6.46 (s, 1H), 6.90-6.93 (d, J = 9.0 Hz, 2H), 7.17-7.32 (m, 5H), 7.47-7.50 (d, J = 9.0 Hz, 2H). ^13^C-NMR (75 MHz, CDCl_3_): δ 52.2, 53.3, 55.5, 62.3, 65.4, 114.6, 118.8, 127.2, 127.4, 127.6, 128.8, 130.4, 135.0, 138.8, 156.3, 165.3, 166.1. HRMS (ESI-TOF): Calcd. for C_21_H_22_NO_6_ [M + H]^+^ 384.1447, Found 384.1449.

**Compound 3:** White solid (72 %), Mp: 117-119 ºC; IR (KBr): 3225, 2361, 1720 cm^-1^; ^1^H NMR (300 MHz, CDCl_3_): δ 3.43-3.45 (d, J = 4.2 Hz, 1H,), 3.78 (s, 3H), 4.30 (s, 1H), 4.45 (s, 1H), 4.84 (s, 1H), 6.10 (s, 1H), 6.25 (s, 1H), 6.87-6.90 (d, J = 8.7 Hz, 2H), 7.22-7.25 (d, J = 7.5 Hz, 2H), 7.28-7.35 (m, 4H). ^13^C-NMR (75 MHz, CDCl_3_): δ 55.5, 56.4, 62.7, 72.7, 114.3, 120.5, 122.7, 127.5, 128.0, 129.1, 130.2, 133.0, 133.8, 156.9, 165.3. HRMS (ESI-TOF): Calcd for C_20_H_19_N_2_O_3_ [M + H]^+^ 335.1396, Found 335.1392.

**General Procedure for the Synthesis of Fused Pyrrolidine/Pyrrolizidine Derivatives**

Sarcosine**5**or proline**8** (1.0 mmol) and isatin4/acenapthequinone10/ninhydrin 14(1.0 mmol) were added to a solution of BHA azetidin-2-yl methyl acrylate 2a,b/ azetidin-2-yl acrylonitrile 3 (1.0 mmol) and the mixture was heated at reflux in methanol (20 ml). After completion of the reaction was indicated by TLC analysis, methanol was evaporated under reduced pressure and the residue diluted with dichloromethane and washed with brine and water. The organic layer was separated and removed and the residue was subjected to column chromatography by using ethyl acetate/hexane as eluent [14].

**Compound 6a:** White solid. (85%), Mp: 239-242 ºC; IR (KBr): 3140, 1765, 1759, 1711 cm^-1^; ^1^H NMR (300 MHz, CDCl_3_): δ 2.39-2.47 (m, 1H), 2.54 (s, -NCH_3_, 3H), 2.58-2.64 (m, 1H), 2.67-2.77 (dd, J = 7.8, 8.7 Hz, 1H), 3.05-3.11 (dd, J = 8.7, 9.3 Hz, 1H), 3.71 (s, -OCH_3_, 3H), 4.71 (s, 1H), 4.92-4.93 (d, J = 1.2 Hz, 1H), 5.54-5.55 (d, J = 0.9 Hz, 1H), 6.79-6.82 (d, J = 8.7 Hz, 2H), 6.95-6.97 (d, J = 7.8 Hz, 1H), 7.07-7.18 (m, 4H), 7.24-7.27 (m, 2H), 7.34-7.41 (m, 3H), 7.72-7.75 (d, J = 7.5 Hz, 1H), 8.86 (s, 1H, N-H). ^13^C NMR (75 MHz, CDCl_3_): δ 28.1, 34.0, 52.9, 55.4, 56.3, 59.1, 71.7, 73.8, 80.0, 114.1, 114.8, 116.5, 120.0, 123.0, 124.2, 127.9, 129.8, 131.0, 135.9, 157.2, 157.3, 162.1, 170.3, 172.4. DEPT-135 NMR (75 MHz, CDCl_3_): δ 28.1, 34.0, 52.9, 55.4, 59.1, 73.8, 80.0, 114.8, 116.5, 116.6, 120.0, 123.0, 124.2, 129.8, 131.0. Mass: m/z 525.60 (M^+^). Anal.Calcd. For C_30_H_27_N_3_O_6_: C, 68.56, H, 5.18, N, 8.00 %; Found: C, 68.65, H, 5.12, N, 7.91%.

CCDC-882505 (for 6a) contains the supplementary crystallographicdata for this paper. These data can be obtained free of chargefrom The Cambridge Crystallographic Data Centre via[www.ccdc.cam.ac.uk/data_request/cif](http://www.ccdc.cam.ac.uk/data_request/cif)

**Compound 6b:** White solid. (81%), Mp: 278-281 ºC; IR (KBr): 3107, 1759, 1744, 1707 cm^-1^; ^1^H NMR (300 MHz, CDCl_3_+ DMSO-d_6_): δ 1.93-2.04 (m, 1H), 2.48 (s, 3H), 2.64-2.72 (m, 2H), 2.97-3.06 (m, 1H), 3.77 (s, 3H), 4.33-4.36 (dd, J = 2.4 Hz, 1H), 4.49-4.52 (d, J = 7.8 Hz, 1H), 4.76-4.77 (d, J = 2.4 Hz, 1H), 6.85-6.88 (d, J = 9.0 Hz, 2H), 7.05-7.12 (m, 2H), 7.32-7.41 (m, 4H), 7.48-7.57 (m, 5H), 10.57 (s, 1H, NH). ^13^C NMR (75 MHz, CDCl3+ DMSO-d6): δ 32.9, 39.0, 57.7, 59.8, 60.2, 60.8, 63.5, 76.5, 79.4, 118.4, 118.8, 119.6, 121.6, 124.2, 125.3, 127.8, 132.7, 134.0, 135.4, 138.4, 142.1, 161.5, 173.9, 177.6. DEPT-135 NMR (75 MHz, CDCl_3_+ DMSO-d_6_): δ 32.9, 39.0, 57.7, 59.8, 60.2, 63.4, 79.4, 118.8, 119.6, 121.6, 124.2, 125.3, 127.8, 132.7, 134.0, 135.4. Mass: m/z 509.62 (M^+^). Anal.Calcd. For C_30_H_27_N_3_O_5_: C, 70.71; H, 5.34; N, 8.25 %; Found: C, 70.65, H, 5.42, N, 8.33%.

**Compound 7:** Brown solid. (83%), Mp: 217-220 ºC; IR (KBr): 3212, 1762, 1744, 1690 cm^-1^; ^1^H NMR (300 MHz, CDCl_3_): δ 2.40-2.47 (m, 1H), 2.56 (s, -NCH_3_, 3H), 3.04-3.11 (m, 1H), 3.73 (s, 3H), 4.69 (s, 1H), 4.92-4.93 (d, J = 1.5 Hz, 1H), 5.53-5.54 (d, J = 1.2 Hz, 1H), 6.81-6.84 (dd, J = 2.4, 2.1 Hz, 2H), 6.90-6.95 (m, 1H), 7.07-7.13 (m, 2H), 7.14-7.15 (m, 1H), 7.17-7.18 (m, 1H), 7.24 (s, 1H), 7.26 (s, 1H), 7.32-7.39 (m, 2H), 7.46-7.50 (dd, J = 2.7 Hz, 1H), 8.70 (s, 1H, N-H). ^13^C NMR (75 MHz, CDCl_3_): δ 28.2, 34.0, 53.0, 55.4, 56.0, 59.0, 71.5, 73.8, 79.9, 114.9, 115.7-115.8 (J = 29.7 Hz), 116.4, 116.6-116.9 (J = 97.2 Hz), 117.5-117.6 (J = 31.5 Hz), 117.9-118.2 (J = 92.7 Hz), 120.1, 123.0, 127.9, 129.8, 132.13-132.16 (J = 8.1 Hz), 157.3, 160.4, 162.0, 169.5, 172.0. DEPT 135 NMR (75 MHz, CDCl_3_): δ 28.2, 34.0, 53.0, 55.4, 59.0, 73.8, 79.9, 114.9, 116.4, 116.6-116.9 (J = 97.2 Hz), 117.5-117.6 (J = 31.5 Hz), 117.9-118.2 (J = 92.7 Hz), 120.1, 123.0, 129.8. Mass: m/z 527.48 (M^+^). Anal.Calcd. For C_30_H_26_FN_3_O_5_: C, 68.30, H, 4.97, N, 7.97 %; Found: C, 68.37, H, 4.90, N, 8.03%.

**Compound 9:** White solid. (79%), M.p: 205-207 ºC; IR (KBr): 3231, 1759, 1746, 1720 cm^-1^; ^1^H NMR (300 MHz, CDCl_3_+ DMSO-d_6_): δ 1.39-1.49 (m, 1H), 1.63-1.78 (m, 2H), 2.04-2.14 (m, 2H), 2.56-2.58 (m, 2H), 2.64-2.70 (m, 2H), 3.75 (s, 3H), 4.46-4.48 (d, J = 6.6 Hz, 1H), 4.56-4.58 (d, J = 7.8 Hz, 1H), 5.70 (s, 1H), 6.84-6.86 (d, J = 6.9 Hz, 2H), 7.02-7.11 (m, 3H), 7.25-7.34 (m, 5H), 7.43-7.46 (d, J = 7.5 Hz, 2H), 7.65-7.67 (d, J = 7.2 Hz, 1H), 10.68 (s, 1H, NH). ^13^C NMR (75 MHz, CDCl_3_+ DMSO-d_6_): δ 29.9, 37.0, 38.7, 54.1, 60.1, 61.6, 67.1, 68.6, 80.9, 84.9, 85.0, 118.8, 118.9, 121.1, 121.4, 125.4, 127.3, 128.7, 133.3, 134.0, 134.2, 135.6, 142.6, 161.7, 162.3, 167.9, 173.2, 179.6. DEPT 135 NMR (75 MHz, CDCl_3_+ DMSO-d_6_): δ 30.0, 37.1, 38.7, 54.1, 60.1, 67.1, 68.6, 84.9, 84.9, 118.9, 121.1, 121.3, 125.4, 127.2, 128.7, 133.3, 134.2, 135.6. Mass: m/z 551.65 (M^+^). Anal.Calcd. For C_32_H_29_N_3_O_6_: C, 69.68, H, 5.30, N, 7.62%; Found: C, 69.75, H, 5.38, N, 7.55%.

**Compound 11a:** Pale yellow solid. (85%), M.p: 181-183 ºC; IR (KBr): 3136, 1759, 1728 cm^-1^; ^1^H NMR (300 MHz, CDCl_3_): δ 2.08-2.18 (m, 1H), 2.22-2.27 (m, 1H), 2.31 (s, 3H), 3.46-3.51 (dd, J = 7.8, 8.1 Hz, 1H), 3.57 (s, 3H), 3.70 (s, 3H), 4.23 (s, 1H), 4.27 (s, 1H), 5.70-5.71 (d, J = 1.2 Hz, 1H), 6.25 (s, 1H), 6.70-6.73 (d, J = 9.0 Hz, 2H), 7.04-7.08 (t, J = 6.9, 7.2 Hz, 1H), 7. 16-7.23 (m, 4H), 7.32-7.37 (t, J = 7.8, 7.5 Hz, 2H), 7.48-7.60 (m, 3H), 7.70-7.79 (t, J = 8.7, 9.0 Hz, 2H). ^13^C NMR (75 MHz, CDCl_3_): δ 28.7, 38.5, 52.1, 55.3, 58.1, 59.7, 66.1, 71.2, 80.4, 87.9, 108.8, 114.3, 116.3, 120.1, 120.5, 122.5, 122.6, 125.7, 125.9, 127.2, 128.8, 129.0, 129.6, 130.8, 134.2, 137.4, 138.8, 156.7, 157.7, 163.4, 172.0. DEPT-135 NMR (75 MHz, CDCl_3_): δ 28.6, 38.5, 52.2, 55.3, 58.1, 59.7, 71.2, 80.3, 113.9, 114.3, 116.3, 116.6, 120.1, 120.5, 122.5, 122.6, 125.7, 125.9, 127.2, 128.8, 129.6. Mass: m/z 592.59 (M^+^). Anal.Calcd. For C_35_H_32_N_2_O_7_: C, 70.93, H, 5.44, N, 4.73%; Found: C, 71.05, H, 5.37, N, 4.80 %.

**Compound 11b:** Pale yellow solid. (81%), Mp: 172-174 ºC; IR (KBr): 3148, 1755, 1728 cm^-1^; ^1^H NMR (300 MHz, CDCl_3_): δ 1.94-2.00 (m, 1H), 2.15-2.25 (m, 1H), 2.33 (s, 3H), 3.31 (s, 3H), 3.48-3.53 (m, 1H), 3.57-3.65 (m, 1H), 3.71 (s, 3H), 4.11-4.13 (t, J = 2.7 Hz, 1H), 4.17-4.18 (d, J = 3.0 Hz, 1H), 4.69-4.70 (d, J = 2.1 Hz, 1H), 6.29 (br s, 1H), 6.72-6.75 (dd, J = 2.1 Hz, 2H), 7.10-7.12 (d, J = 6.9 Hz, 1H), 7.19-7.22 (dd, J = 2.1 Hz, 2H), 7.31-7.35 (m, 5H), 7.45-7.50 (dd, J = 7.2 Hz, 1H), 7.54-7.59 (dd, J = 6.9 Hz 1H), 7.65-7.67 (d, J = 6.3 Hz, 1H), 7.75-7.78 (dd, J = 1.8, 2.1 Hz, 2H). ^13^C NMR (75 MHz, CDCl_3_): δ 29.1, 38.6, 51.9, 55.4, 56.4, 58.1, 59.8, 66.2, 73.76, 88.4, 108.38, 114.3, 119.9, 120.5, 122.4, 125.7, 125.8, 127.1, 127.7, 127.9, 128.8, 128.9, 129.8, 130.8, 134.4, 134.7, 137.4, 139.0, 156.4, 165.6, 171.9. DEPT-135 NMR (75 MHz, CDCl_3_): δ 29.1, 38.6, 51.9, 55.4, 56.3, 58.1, 59.8, 73.7, 114.3, 119.9, 120.5, 122.4, 125.7, 125.8, 127.1, 127.7, 127.9, 128.8, 128.9. Mass: m/z 576.70 (M^+^). Anal.Calcd. For C_35_H_32_N_2_O_6_: C, 72.90; H, 5.59; N, 4.86 %; Found: C, 72.82, H, 5.67, N, 4.95 %.

**Compound 12:** Pale yellow solid. (84%), Mp: 248-250 ºC; IR (KBr): 3163, 2361, 1728 cm^-1^; ^1^H NMR (300 MHz, CDCl_3_): δ 2.08 (s, 3H), 2.43-2.51 (m, 1H), 2.96-3.06 (dt, J = 4.8, 5.4 Hz, 1H), 3.36-3.44 (dt, J = 5.1 Hz, 1H), 3.50-3.58 (dt, J = 4.2 Hz, 1H), 3.76 (s, 3H), 4.48-4.49 (d, J = 2.1 Hz, 1H), 4.79 (s, 1H), 4.95-4.96 (d, J = 1.8 Hz, 1H), 6.62 (br s, 1H), 6.78-6.81 (d, J = 8.7 Hz, 2H), 7.22-7.25 (d, J = 9.0 Hz, 2H), 7.32-7.39 (m, 5H), 7.60-7.65 (t, J = 7.8 Hz 1H), 7.75-7.82 (m, 2H), 7.96-8.01 (t, J = 8.4, 7.2 Hz 2H), 8.18-8.21 (d, J = 8.1 Hz, 1H). ^13^C NMR (75 MHz, CDCl_3_): δ 27.7, 35.2, 47.4, 51.3, 54.5, 55.5, 61.6, 69.0, 77.2, 81.3, 114.5, 118.6, 119.5, 122.0, 123.7, 127.1, 127.9, 128.0, 128.7, 129.2, 129.6, 130.6, 130.9, 131.1, 132.6, 134.1, 143.6, 156.4, 165.4. Mass: m/z 543.57 (M^+^). Anal.Calcd. For C_34_H_29_N_3_O_4_: C, 75.12; H, 5.38; N, 7.73 %; Found: C, 75.19, H, 5.30, N, 7.81 %.

**Compound 13a:** Yellow solid. (80%), M.p: δ 185-187 ºC; IR (KBr): 3148, 1751, 1720 cm^-1^; ^1^H NMR (300 MHz, CDCl_3_): δ 1.78-1.93 (m, 2H), 2.08-2.15 (m, 2H), 2.37-2.44 (dd, J = 7.5, 7.8 Hz, 1H), 2.49-2.56 (dd, J = 8.7, 9.0 Hz, 1H), 3.00-3.05 (t, J = 6.6 Hz, 1H), 3.38 (s, 3H), 3.57-3.65 (m, 1H), 3.71 (s, 3H), 3.78-3.84 (m, 1H), 4.28 (s, 1H), 4.37 (s, 1H), 5.69 (s, 1H), 6.70-6.73 (d, J = 8.7 Hz, 2H), 7.04-7.09 (t, J = 7.2, 6.9 Hz, 1H), 7.16-7.19 (d, J = 8.7 Hz, 2H), 7.23-7.25 (m, 2H), 7.32-7.37 (t, J = 6.0, 7.2 Hz, 3H), 7.46-7.51 (m, 3H), 7.71-7.78 (dd, J = 8.1, 6.6 Hz, 2H). ^13^ C NMR (75 MHz, CDCl_3_): δ 26.8, 31.0, 36.6, 49.5, 52.2, 55.3, 60.0, 66.6, 68.2, 73.1, 80.6, 89.6, 109.2, 114.3, 116.5, 119.8, 120.1, 122.6, 123.4, 125.6, 126.1, 127.2, 128.7, 129.0, 129.6, 130.7, 135.0, 137.1, 139.4, 156.7, 157.8, 163.2, 172.9. DEPT-135 NMR (75 MHz, CDCl_3_): δ 26.9, 31.0, 36.6, 49.5, 52.3, 55.3, 60.0, 66.6, 73.1, 80.6, 114.3, 116.5, 119.8, 120.1, 122.6, 123.4, 125.6, 126.1, 127.2, 128.7, 129.6. Mass: m/z 618.61 (M^+^). Anal.Calcd. For C_37_H_34_N_2_O_7_: C, 71.83, H, 5.54, N, 4.53%; Found: C, 71.92, H, 5.62, N, 4.46%

**Compound 13b:** Pale yellow solid. (83%), M.p: 256-258ºC; IR (KBr): 3194, 1759, 1721 cm^-1^; ^1^H NMR (300 MHz, CDCl_3_): δ 1.87-2.00 (m, 2H), 2.10-2.20 (m, 1H), 2.18-2.25 (m, 2H), 2.35-2.42 (dd, J = 7.8 Hz, 1H), 2.99-3.40 (m, 1H), 3.11 (s, 3H), 3.52-3.60 (dd, J = 8.7, 9.0 Hz, 1H), 3.72 (s, 3H), 3.96-4.03 (dd, J = 7.5, 6.9 Hz, 1H), 4.12 (s, 1H), 4.28-4.29 (d, J = 3.0 Hz, 1H), 4.67 (s, 1H), 6.72-6.75 (d, J = 8.7 Hz, 2H), 7.16-7.19 (d, J = 8.7 Hz, 2H), 7.25-7.36 (m, 6H), 7.44-7.49 (t, J = 7.5, 7.8 Hz, 1H), 7.53-7.58 (m, 2H), 7.73-7.78 (m, 2H). ^13^C NMR (75 MHz, CDCl_3_): δ 26.9, 31.2, 36.4, 49.5, 51.9, 55.4, 56.7, 60.2, 66.7, 68.3, 75.6, 90.3, 108.8, 114.3, 119.8, 123.1, 125.6, 126.0, 127.1, 127.8, 127.9, 128.8, 129.0, 129.7, 130.8, 134.8, 135.1, 137.1, 139.6, 156.4, 165.4, 172.9. DEPT-135 NMR (75 MHz, CDCl_3_): δ 26.9, 31.2, 36.4, 49.5, 51.9, 55.4, 56.7, 60.2, 66.7, 75.6, 114.2, 119.8, 119.9, 123.1, 125.6, 126.1, 127.1, 127.8, 127.9, 128.8, 129.0. Mass: m/z 602.62 (M^+^). Anal.Calcd. For C_37_H_34_N_2_O_6_: C, 73.74; H, 5.69; N, 4.65 %; Found: C, 73.83, H, 5.60, N, 4.74%.

**Compound 14:** Pale yellow solid. (80%), M.p: 196-198 ºC; IR (KBr): 3202, 2353, 1721 cm^-1^; ^1^H NMR (300 MHz, CDCl_3_): δ 1.86-2.00 (m, 2H), 2.07-2.12 (m, 2H), 2.18-2.21 (m, 1H), 2.40-2.47 (m, 1H), 2.93-3.02 (m, 1H), 3.06-3.15 (m, 1H), 3.71 (s, 3H), 3.78 (s, 1H), 3.82-3.85 (m, 1H), 4.32 (s, 1H), 4.79 (s, 1H), 6.73-6.77 (m, 2H), 7.18-7.21 (m, 2H), 7.26-7.37 (m, 5H), 7.48-7.51 (m, 1H), 7.57-7.70 (m, 3H), 7.79-7.83 (m, 1H), 7.87-7.90 (m, 1H). ^13^C NMR (75 MHz, CDCl_3_): δ 26.5, 31.0, 32.9, 49.3, 53.5, 55.4, 55.8, 59.4, 67.9, 75.3, 89.5, 110.0, 114.5, 120.4, 120.7, 124.4, 126.0, 126.9, 127.8, 127.9, 128.1, 129.0, 129.1, 129.2, 130.8, 132.9, 133.9, 137.3, 137.6, 156.9, 165.3. DEPT-135 NMR (75 MHz, CDCl_3_): δ 26.5, 31.0, 32.8, 49.3, 55.4, 55.7, 59.3, 67.9, 75.2, 114.5, 120.4, 120.6, 124.4, 126.0, 126.8, 127.8, 127.9, 128.1, 129.1, 129.2. Mass: m/z 569.70 (M^+^). Anal.Calcd. For C_36_H_31_N_3_O_4_: C, 75.90; H, 5.49; N, 7.38 %; Found: C, 75.83, H, 5.58, N, 7.45%.

**Compound 16a:** Colorless solid. (85%), M.p: 172-174 ºC; IR (KBr): 3186, 1758, 1751, 1713 cm^-1^; ^1^H NMR (300 MHz, CDCl_3_): δ 1.94-2.02 (m, 1H), 2.30-2.36 (m, 1H), 2.40 (s, 3H), 3.28-3.34 (dd, J = 7.2, 8.1 Hz, 1H), 3.42-3.49 (m, J = 8.7, 7.8 Hz, 1H), 3.71 (s, 6H), 4.18 (s, 1H), 4.31 (s, 1H), 5.61 (s, 1H), 6.75-6.78 (d, J = 8.7 Hz, 2H), 7.03-7.08 (t, J = 7.2, 6.9 Hz, 1H), 7.15-7.17 (d, J = 8.7 Hz, 2H), 7.21-7.25 (m, 2H), 7.31-7.36 (t, J = 7.8, 7.5 Hz, 2H), 7.47-7.51 (t, J = 6.9 Hz, 1H), 7.64-7.70 (m, 3H). ^13^C NMR (75 MHz, CDCl_3_): δ 28.4, 37.6, 52.9, 55.4, 58.2, 59.2, 67.0, 72.2, 80.5, 85.3, 103.2, 114.4, 116.3, 120.1, 122.6, 122.7, 125.1, 128.8, 129.6, 130.7, 136.4, 137.1, 149.2, 156.9, 157.6, 161.1, 171.1, 198.2. DEPT-135 NMR (75 MHz, CDCl_3_): δ 28.4, 37.6, 52.9, 55.4, 58.2, 59.1, 72.2, 80.4, 114.4, 116.3, 120.1, 122.6, 122.7, 125.1, 129.6, 130.7, 137.1. Mass: m/z 570.64 (M^+^). Anal.Calcd. For C_32_H_30_N_2_O_8_: C, 67.36, H, 5.30, N, 4.91%; Found: C, 67.43, H, 5.39, N, 4.82 %.

**Compound 16b:** Colorless solid. (79%), M.p: 172-174 ºC; IR (KBr): 3116, 1759, 1752, 1721 cm^-1^; ^1^H NMR (300 MHz, CDCl_3_): δ 2.01-2.06 (m, 1H), 2.47 (s, 3H), 3.33-3.43 (m, 2H), 3.50 (s, 3H), 3.75 (s, 3H), 4.05 (s, 1H), 4.23-4.24 (d, J = 3.3 Hz, 1H), 4.57 (s, 1H), 5.66 (br s, 1H), 6.78-6.80 (d, J = 8.7 Hz, 2H), 7.18-7.21 (d, J = 8.7 Hz, 2H), 7.29-7.37 (m, 5H), 7.50-7.55 (t, J = 7.2, 7.5 Hz ,1H), 7.68-7.76 (m, 2H), 7.80-7.82 (d, J = 7.5 Hz, 1H). ^13^ C NMR (75 MHz, CDCl_3_): δ 28.9, 37.7, 52.6, 55.4, 56.6, 58.2, 59.4, 67.1, 75.1, 85.8, 102.8, 114.3, 120.2, 122.7, 125.1, 127.8, 129.0, 129.4, 130.7, 134.3, 136.6, 137.1, 149.3, 156.7, 165.3, 170.8, 198.4. DEPT-135 NMR (75 MHz, CDCl_3_): δ 28.9, 37.7, 52.6, 55.4, 56.6, 58.2, 59.4, 75.1, 77.2, 114.3, 120.2, 122.7, 125.1, 127.8, 129.0, 130.7, 137.1. Mass: m/z 554.51 (M^+^). Anal.Calcd. For C_32_H_30_N_2_O_7_: C, 69.30, H, 5.45, N, 5.05 %; Found: C, 69.37, H, 5.39, N, 4.97 %.

**Compound 17:** White solid. (83%), M.p: 132-134 ºC; IR (KBr): 3163, 2355, 1759, 1720 cm-1; ^1^H NMR (300 MHz, CDCl_3_): δ 1.92-2.02 (m, 1H), 2.31-2.36 (dd, J = 4.2, 3.9 Hz, 1H,), 2.41(s, 3H), 3.30-3.35 (t, J = 7.5 Hz, 1H), 3.43-3.48 (m, 1H), 3.73 (s, 3H), 4.17 (s, 1H), 4.31 (s, 1H), 5.61 (s, 1H), 6.76-6.78 (d, J = 8.7 Hz, 2H), 7.04-7.09 (t, J = 6.9 Hz, 1H), 7.15-7.18 (d, J = 8.4 Hz, 2H), 7.21-7.23 (d, J = 7.8 Hz, 2H), 7.32-7.37 (t, J = 7.2, 7.5 Hz, 2H), 7.48-7.52 (m, 1H), 7.64-7.76 (m, 3H). ^13^C NMR (75 MHz, CDCl_3_): δ 23.4, 37.6, 52.9, 55.4, 58.2, 59.1, 67.0, 72.2, 80.5, 85.3, 103.2, 114.4, 116.3, 120.1, 122.6, 122.7, 125.1, 128.8, 129.6, 130.7, 136.4, 137.1, 149.2, 156.9, 157.6, 163.1, 171.2, 198.2. DEPT-135 NMR (75 MHz, CDCl_3_): δ 28.4, 37.6, 52.9, 55.4, 58.2, 59.1, 72.2, 80.5, 114.4, 116.3, 120.1, 122.6, 122.7, 125.1, 129.6, 130.7, 137.1. Mass: m/z 521.61(M^+^). Anal.Calcd. For C_31_H_27_N3O_5_: C, 71.39; H, 5.22; N, 8.06%; Found: C, 71.46, H, 5.30, N, 7.78%.

**Compound 18a:** Colorless solid. (82%), M.p: 167-169 ºC; IR (KBr): 3163, 1759, 1750, 1720 cm^-1^; ^1^H NMR (300 MHz, CDCl_3_): δ 1.74-1.86 (m, 3H), 1.89-2.01 (m, 1H), 2.04-2.10 (m, 1H), 2.24-2.31 (dd, J = 7.5 Hz, 1H), 2.44-2.51 (dd, J = 7.2, 7.5 Hz, 1H), 2.85-2.90 (m, 1H), 3.46-3.55 (m, 1H), 3.67 (s, 3H), 3.74 (s, 3H), 4.23 (s, 1H), 4.34 (s, 1H), 5.62 (s, 1H), 6.75-6.78 (d, J = 9.0 Hz, 2H), 7.04-7.09 (t, J = 7.2 Hz, 1H), 7.15-7.23 (m, 4H), 7.32-7.37 (m, 2H), 7.45-7.49 (m, 1H), 7.61 (s, 1H), 7.63-7.68 (t, J = 7.5 Hz, 2H). ^13^C NMR (75 MHz, CDCl_3_): δ 26.5, 30.3, 34.7, 48.3, 52.9, 55.4, 59.4, 67.8, 73.7, 77.2, 80.5, 86.5, 104.5, 114.4, 116.4, 120.1, 122.7, 122.9, 124.4, 128.7, 129.6, 130.5, 136.4, 137.0, 149.1, 156.8, 157.6, 162.9, 171.8, 197.0. DEPT-135 NMR (75 MHz, CDCl_3_): δ 26.5, 30.3, 34.7, 48.3, 53.0, 55.4, 59.4, 67.8, 73.7, 80.5, 114.3, 116.4, 120.1, 122.7, 122.9, 124.4, 129.6, 130.6, 137.0. Mass: m/z 596.70 (M^+^). Anal.Calcd. For C_34_H_32_N_2_O_8_: C, 68.45, H, 5.41, N, 4.70%; Found: C, 68.58, H, 5.49, N, 4.76 %.

CCDC-882507 (for 18a) contains the supplementary crystallographic data for this paper. These data can be obtained free of charge from The Cambridge Crystallographic Data Centre via [www.ccdc.cam.ac.uk/data_request/cif](http://www.ccdc.cam.ac.uk/data_request/cif).

**Compound 18b:** Colorless solid. (84%), M.p: 188-191 ºC; IR (KBr): 3146, 1762, 1755, 1728 cm^-1^; ^1^H NMR (300 MHz, CDCl_3_): δ 1.74-1.89 (m, 2H), 2.01-2.08 (m, 2H), 2.10-2.15 (dd, J = 7.8 Hz, 1H), 2.28-2.35 (dd, J = 6.6 Hz, 1H), 2.84-2.91 (m, 1H), 3.46 (s, 3H), 3.75 (s, 3H), 3.81-3.85 (t, J = 6.9, 6.6 Hz, 1H), 4.07-4.09 (t, J = 2.4, 3.0 Hz 1H), 4.26-4.27 (d, J = 3.3 Hz, 1H), 4.60-4.61 (d, J = 2.1 Hz, 1H), 6.76-6.79 (d, J = 9.0 Hz, 2H), 7.16-7.19 (d, J = 9.0 Hz, 2H), 7.29-7.38 (m, 5H), 7.46-7.51 (m, 1H), 7.65-7.70 (m, 3H). ^13^C NMR (75 MHz, CDCl_3_): δ 26.6, 30.5, 34.5, 48.4, 52.6, 55.4, 56.6, 59.6, 67.8, 68.0, 76.3, 87.0, 103.7, 114.3, 120.0, 122.8, 124.5, 127.8, 127.9, 129.0, 129.3, 130.5, 134.4, 136.4, 137.1, 149.3, 156.6, 165.2, 171.6, 197.2. DEPT-135 NMR (75 MHz, CDCl_3_): δ 26.6, 30.5, 34.5, 48.4, 52.7, 55.4, 56.6, 59.6, 68.0, 76.3, 114.3, 120.0, 122.9, 124.5, 127.8, 127.9, 129.1, 130.6, 137.1. Mass: m/z 580.71 (M^+^). Anal.Calcd. For C_34_H_32_N_2_O_7_: C, 70.33; H, 5.56; N, 4.82 %; Found: C, 70.24, H, 5.63, N, 4.75 %.

**Compound 19:** White solid. (81%), M.p: 132-134 ºC; IR (KBr): 3140, 2352, 1758, 1716 cm^-1^; ^1^H NMR (300 MHz, CDCl_3_): δ 1.77-1.91 (m, 2H), 1.96-2.01 (m, 2H), 2.27-2.33 (dd, J = 6.6 Hz, 2H), 2.96-3.04 (m, 1H), 3.10-3.19 (m, 1H), 3.69-3.73 (m, 1H), 3.75 (s, 3H), 3.86 (s, 1H), 4.32 (s, 1H), 4.74-4.75 (d, J = 2.1 Hz, 1H), 6.17 (br s, 1H), 6.78-6.81 (d, J = 9.0 Hz, 2H), 7.21-7.24 (d, J = 9.0 Hz, 2H), 7.26-7.39 (m, 5H), 7.52-7.57 (m, 1H), 7.72-7.74 (m, 2H), 7.79-7.82 (d, J = 7.5 Hz, 1H). ^13^C NMR (75 MHz, CDCl_3_): δ 26.4, 30.2, 34.6, 49.5, 52.9, 55.4, 55.7, 58.9, 68.5, 74.9, 86.0, 104.4, 114.6, 119.2, 120.1, 123.6, 124.9, 127.8, 128.3, 128.8, 129.3, 131.0, 133.6, 135.7, 137.8, 148.2, 156.9, 164.9, 195.8. DEPT-135 NMR (75 MHz, CDCl_3_): δ 26.4, 30.2, 34.5, 49.5, 55.4, 55.7, 58.9, 68.5, 74.9, 114.6, 120.1, 123.6, 124.9, 127.8, 128.3, 129.3, 131.0, 137.8. Mass: m/z 547.66 (M^+^). Anal.Calcd. For C_33_H_29_N_3_O_5_: C, 72.38; H, 5.34; N, 7.67%; Found: C, 72.45, H, 5.29, N, 7.73%.

CCDC-885084 (for 19) contains the supplementary crystallographic data for this paper. These data can be obtained free of charge from The Cambridge Crystallographic Data Centre via [www.ccdc.cam.ac.uk/data_request/cif](http://www.ccdc.cam.ac.uk/data_request/cif).

**Result:**

S1 Fig. X-ray crystal structure of compound 6a

(a) S. Sundaramoorthy, R. Rajesh, R. Raghunathan, D. Velmurugan, *Acta Cryst.* **2012**, *E68*, o2202; (b) CCDC-882505 (for **6a**) contains the supplementary crystallographic data for this paper. These data can be obtained free of charge from The Cambridge Crystallographic Data Centre via www.ccdc.cam.ac.uk/data_request/cif.

S2 Fig. X-ray crystal structure of compound 18a

S. Sundaramoorthy, R. Rajesh, R. Raghunathan, D. Velmurugan, *Acta Cryst.* **2012**, *E68*, o2200- o2201; (b) CCDC-882507 (for **18a**) contains the supplementary crystallographic data for this paper. These data can be obtained free of charge from The Cambridge Crystallographic Data Centre via [www.ccdc.cam.ac.uk/data_request/cif](http://www.ccdc.cam.ac.uk/data_request/cif)

S3 Fig. X-ray crystal structure of compound 19

CCDC-885084 (for **19**) contains the supplementary crystallographic data for this paper. These data can be obtained free of charge from The Cambridge Crystallographic Data Centre via www.ccdc.cam.ac.uk/data_request/cif

S4 Fig. 2D representations of *β*-lactam compounds. A) Ampicillin B) Compound 7 C) Compound 6a and D) Compound 3 to the active site pocket of PBP (PDB ID: 2Z2M.
