# Supplementary material for: Bioactivity Studies of β-Lactam Derived Polycyclic Fused Pyrroli-Dine/Pyrrolizidine Derivatives in Dentistry: In Vitro, In Vivo and In Silico Studies
Source: PLoS One. 2015 Jul 17;10(7):e0131433. doi: 10.1371/journal.pone.0131433 (PMC4505899; doi:10.1371/journal.pone.0131433)
Supplement: S1 Table — (DOCX) [file pone.0131433.s006.docx]

**S1 Table.** Zone of inhibition (in mm) of *β*-lactams against pathogens in root canal infection.

| S.No | Compound name/ organisms | *E. faecalis* (ATCC) | *E. faecalis* (RS1) | *E. faecalis* (RS2) | *E. faecalis* (RS3) | *E. faecalis* (RS4) | *E. faecalis* (RS5) | *Streptococcus* sp | *S. aureus* |
| --- | --- | --- | --- | --- | --- | --- | --- | --- | --- |
|  |  |  |  |  |  |  |  |  |  |
| 1 | 2a | 16 | 19 | - | - | 15 | 8 | 17 | 13 |
| 2 | 3 | 18 | 19 | 10 | 8 | 9 | 7 | 18 | 14 |
| 3 | 6a | 17 | 19 | 10 | 7 | 11 | 7 | 18 | 13 |
| 4 | 7 | 17 | 18 | 9 | 14 | 10 | 13 | 15 | 13 |
| 5 | 9 | 16 | 18 | 10 | - | 15 | - | - | - |
| 6 | 11a | - | - | 9 | 10 | - | 7 | - | - |
| 7 | 11b | 20 | 17 | 8 | - | 16 | 8 | 15 | 15 |
| 8 | 12 | 18 | 15 | 7 | 9 | 14 | 8 | 16 | 15 |
| 9 | 13a | 18 | 17 | 10 | - | 19 | - | 16 | - |
| 10 | 13b | 17 | 18 | 8 | 8 | - | 10 | 14 | 14 |
| 11 | 14 | 17 | 17 | - | 8 | 11 | - | 16 | 13 |
| 12 | 16a | - | - | - | - | 16 | 9 | - | 12 |
| 13 | 18a | 16 | 14 | - | - | - | - | 15 | 15 |
| 14 | 18b | 16 | 18 | 10 | 9 | 14 | - | 16 | 8 |
| 15 | 19 | 18 | 16 | 8 | 8 | 13 | 11 | 16 | - |
| RC | Amp | 19 | - | - | - | - | - | 21 | 18 |

-, no activity, Amp- Ampicillin, RS (1-5) - Resistant strains isolated from Root Canal Treatment failure cases.
