# Supplementary material for: Bioactivity Studies of β-Lactam Derived Polycyclic Fused Pyrroli-Dine/Pyrrolizidine Derivatives in Dentistry: In Vitro, In Vivo and In Silico Studies
Source: PLoS One. 2015 Jul 17;10(7):e0131433. doi: 10.1371/journal.pone.0131433 (PMC4505899; doi:10.1371/journal.pone.0131433)
Supplement: S2 Table — (DOCX) [file pone.0131433.s007.docx]

**S2 Table.** Percentage of hemolysis after treatment with *β*-lactam compounds.

| S. No |  | Antibacterial agents |  | % of hemolysis |  | Mean ±SD |
| --- | --- | --- | --- | --- | --- | --- |
| 1 |  | Untreated |  | 0 |  | 0.064± 0.0042 |
| 2 |  | 3 |  | 0. 67 |  | 0.0698± 0.0002 |
| 3 |  | 7 |  | 7.55 |  | 0.121 ± 0.004 |
| 4 |  | 6a |  | 0.9 |  | 0.0725 ± 0.0007 |
| 5 |  | Ampicillin |  | 11.6 |  | 0.164 ± 0.006 |
| 6 |  | 0.1 % DMSO |  | 0 |  | 0.0608 ± 0.002 |
| 7 |  | Triton X-100 |  | 100 |  | 0.9225 ± 0.026 |
