# Supplementary material for: Bioactivity Studies of β-Lactam Derived Polycyclic Fused Pyrroli-Dine/Pyrrolizidine Derivatives in Dentistry: In Vitro, In Vivo and In Silico Studies
Source: PLoS One. 2015 Jul 17;10(7):e0131433. doi: 10.1371/journal.pone.0131433 (PMC4505899; doi:10.1371/journal.pone.0131433)
Supplement: S3 Table — (DOCX) [file pone.0131433.s008.docx]

**S3 Table.** Inhibitory effect of *β -*lactam on direct mutagenicity in *S. typhimurium* TA 98 cells and TA 100 cells without metabolic activation system

| S. No | | Test agents |  | Concentration (µg/ml) | Number of revertants (Mean ±SD) | |
| --- | --- | --- | --- | --- | --- | --- |
|  |  |  |  |  | TA 98 | TA 100 |
| 1 | | Untreated |  | - | 488± 18 | 348±37 |
| 2 | | 3 |  | 250 | 441± 13 | 330±17 |
| 3 | | 7 |  | 250 | 413±19 | 342±24 |
| 4 |  | Ampicillin |  | 250 | 407±43 | 336±32 |
| 5 | | Sodium Azide |  | 2 | ---- | 1403±67 |
| 6 | | Mitomycin C |  | 5 | 1573±102 | ---- |

^---^ indicates no revertant colony formation.
