# Supplementary material for: Bioactivity Studies of β-Lactam Derived Polycyclic Fused Pyrroli-Dine/Pyrrolizidine Derivatives in Dentistry: In Vitro, In Vivo and In Silico Studies
Source: PLoS One. 2015 Jul 17;10(7):e0131433. doi: 10.1371/journal.pone.0131433 (PMC4505899; doi:10.1371/journal.pone.0131433)
Supplement: S4 Table — (DOCX) [file pone.0131433.s009.docx]

**S4 Table.** Wing spot assay after larval treatment with *β*-lactam

| S. No | Compound name | Concentrations |  | Number of spots | Type of spots | *p* value |
| --- | --- | --- | --- | --- | --- | --- |
| 1 | Untreated | - |  | 32 | Single spot mwh with two hairs |  |
| 2 | *β*-lactam LD | 250 |  | 35 | Single, large mwh with two hairs | 0.851 |
| 3 | *β*-lactam HD | 500 |  | 42 | mwh with two hairs | 0.922 |
| 4 | Ampicillin | 250 |  | 45 | mwh with two hairs | 0.920 |
| 5 | 0.1% DMSO | - |  | 32 | mwh with two hairs | 0.947 |
| 6 | EMS | 5mM |  | 268 | Single, large spots and mwh with two hairs, flr with twin spots | 0.036 |
